# Supplementary material for: Comparative analysis of twelve mitogenomes of Caliscelidae (Hemiptera: Fulgoromorpha) and their phylogenetic implications
Source: PeerJ. 2021 Nov 16;9:e12465. doi: 10.7717/peerj.12465 (PMC8603831; doi:10.7717/peerj.12465)
Supplement: Supplemental Information 1 — Figure S1. Secondary structures of the transfer RNAs in the mitogenome of Augilina tetraina. Dashes and dots indicate Watson–Crick and GU base pairing, respectively. Figure S2. Secondary structures of the transfer RNAs in the mitogenome of Augilina triaina. Dashes and dots indicate Watson–Crick and GU base pairing, respectively. Figure S3. Secondary structures of the transfer RNAs in the mitogenome of Symplana brevistrata. Dashes and dots indicate Watson–Crick and GU base pairing, respectively. Figure S4. Secondary structures of the transfer RNAs in the mitogenome of Symplana lii. Dashes and dots indicate Watson–Crick and GU base pairing, respectively. Figure S5. Secondary structures of the transfer RNAs in the mitogenome of Neosymplana vittatum. Dashes and dots indicate Watson–Crick and GU base pairing, respectively. Figure S6. Secondary structures of the transfer RNAs in the mitogenome of Pseudosymplanella nigrifasciata. Dashes and dots indicate Watson–Crick and GU base pairing, respectively. Figure S7. Secondary structures of the transfer RNAs in the mitogenome of Symplanella brevicephala. Dashes and dots indicate Watson–Crick and GU base pairing, respectively. Figure S8. Secondary structures of the transfer RNAs in the mitogenome of Symplanella unipuncta. Dashes and dots indicate Watson–Crick and GU base pairing, respectively. Figure S9. Secondary structures of the transfer RNAs in the mitogenome of Augilodes binghami. Dashes and dots indicate Watson–Crick and GU base pairing, respectively. Figure S10. Secondary structures of the transfer RNAs in the mitogenome of Cylindratus longicephalus. Dashes and dots indicate Watson–Crick and GU base pairing, respectively. Figure S11. Secondary structures of the transfer RNAs in the mitogenome of Caliscelis shandongensis. Dashes and dots indicate Watson–Crick and GU base pairing, respectively. Figure S12. Secondary structures of the transfer RNAs in the mitogenome of Peltonotellus sp. Dashes and dots indicate Watson–Crick [file peerj-09-12465-s001.zip › supplementary materials -figure and table/Figure captions.docx]

**Figure captions**

Figure 1. Circular maps of the12 newly sequenced mitogenomes of Caliscelidae. Protein-coding, ribosomal, and transfer RNA genes are shown with standard abbreviations. Gene orientations are indicated by arrow directions. Protein-coding genes, transfer RNA genes, control regions, and two ribosomal RNA genes are shown in yellow, aubergine, blue, and red, respectively.

Figure 2. Number of codon usages in the protein-coding genes of the12 newly sequenced mitogenomes of Caliscelidae.

Figure 3. Relative synonymous codon usage in the protein-coding genes of the12 newly sequenced mitogenomes of Caliscelidae. Codon families are indicated below the X-axis. The color of the codon family below the X-axis corresponds to the color above the X-axis. The stop codon is not given. Codons absent in mitogenomes are shown at the top of columns.

Figure 4. Phylogenetic trees of Fulgoroidea inferred using MrBayes (Bayesian inference) and maximum likelihood (ML) analysis based on the nucleotide sequences of 13 protein-coding genes. Bayesian posterior probabilities (BPPs) and bootstrap percentages (BPs) are indicated on branches.

Figure 5. Nucleotide diversity (A) and the ratio of Ka/Ks (B) of protein-coding genes from 16 reported Caliscelidae mitogenomes.

**Supplementary Materials:**

Figure S1. Secondary structures of the transfer RNAs in the mitogenome of *Augilina tetraina*. Dashes and dots indicate Watson–Crick and GU base pairing, respectively.

Figure S2. Secondary structures of the transfer RNAs in the mitogenome of *Augilina triaina*. Dashes and dots indicate Watson–Crick and GU base pairing, respectively.

Figure S3. Secondary structures of the transfer RNAs in the mitogenome of *Symplana brevistrata*. Dashes and dots indicate Watson–Crick and GU base pairing, respectively.

Figure S4. Secondary structures of the transfer RNAs in the mitogenome of *Symplana lii*. Dashes and dots indicate Watson–Crick and GU base pairing, respectively.

Figure S5. Secondary structures of the transfer RNAs in the mitogenome of *Neosymplana vittatum*. Dashes and dots indicate Watson–Crick and GU base pairing, respectively.

Figure S6. Secondary structures of the transfer RNAs in the mitogenome of *Pseudosymplanella nigrifasciata*. Dashes and dots indicate Watson–Crick and GU base pairing, respectively.

Figure S7. Secondary structures of the transfer RNAs in the mitogenome of *Symplanella brevicephala*. Dashes and dots indicate Watson–Crick and GU base pairing, respectively.

Figure S8. Secondary structures of the transfer RNAs in the mitogenome of *Symplanella unipuncta*. Dashes and dots indicate Watson–Crick and GU base pairing, respectively.

Figure S9. Secondary structures of the transfer RNAs in the mitogenome of *Augilodes binghami*. Dashes and dots indicate Watson–Crick and GU base pairing, respectively.

Figure S10. Secondary structures of the transfer RNAs in the mitogenome of *Cylindratus longicephalus*. Dashes and dots indicate Watson–Crick and GU base pairing, respectively.

Figure S11. Secondary structures of the transfer RNAs in the mitogenome of *Caliscelis shandongensis*. Dashes and dots indicate Watson–Crick and GU base pairing, respectively.

Figure S12. Secondary structures of the transfer RNAs in the mitogenome of *Peltonotellus* sp. Dashes and dots indicate Watson–Crick and GU base pairing, respectively.

Figure S13. Features of the AT-rich region in the *Augilina tetraina* mitogenome. The tandem repeats are shaded yellow.

Figure S14. Features of the AT-rich region in the *Augilina triaina* mitogenome. The tandem repeats are shaded yellow.

Figure S15. Features of the AT-rich region in the *Symplana brevistrata* mitogenome. The two tandem repeats are shaded yellow and gray, respectively. * indicates a mismatch; - indicates an insertion or deletion.

Figure S16. Features of the AT-rich region in the *Symplana lii* mitogenome. The two tandem repeats are shaded yellow and gray, respectively. * indicates a mismatch; - indicates an insertion or deletion.

Figure S17. Features of the AT-rich region in the *Neosymplana vittatum* mitogenome. The four tandem repeats are shaded blue, gray, yellow, and green, respectively. * indicates a mismatch; - indicates an insertion or deletion.

Figure S18. Features of the AT-rich region in the *Pseudosymplanella nigrifasciata* mitogenome. The two tandem repeats are shaded yellow and gray, respectively.

Figure S19. Features of the AT-rich region in the *Symplanella brevicephala* mitogenome. The two tandem repeats are shaded yellow and gray, respectively. * indicates a mismatch; - indicates an insertion or deletion.

Figure S20. Features of the AT-rich region in the *Symplanella unipuncta* mitogenome. The two tandem repeats are shaded yellow and gray, respectively. * indicates a mismatch; - indicates an insertion or deletion.

Figure S21. Features of the AT-rich region in the *Augilodes binghami* mitogenome. The two tandem repeats are shaded yellow and gray, respectively. * indicates a mismatch.

Figure 22. Features present in the AT-rich region of the *Cylindratus longicephalus* mitogenome. The two tandem repeats are shaded yellow and gray. * indicates a mismatch; - indicates an insertion or deletion.

Figure 23. Features of the AT-rich region in the *Caliscelis shandongensis* mitogenome. The tandem repeats are shaded yellow. * indicates a mismatch.

Figure 24. Features of the AT-rich region in the *Peltonotellus* sp. mitogenome. The two tandem repeats are shaded yellow and gray. * indicates a mismatch.

Table S1 Mitogenomes used in this study

Table S2 The partition schemes and best substitution models used in the phylogenetic analysis.

Table S3 List of total size and intergenic nucleotides for mitochondrial genes of 12 newly sequenced species with lengths of genes, anticodons of tRNAs, and start/stop codons of protein-coding genes.

Table S4 Base composition of 12 mitochondrial whole genome, 13 PCGs, 22 tRNAs, 2 rRNAs, and Control Region.

Table S5 Codon number and RSCU in the 12 mitochondrial PCGs.

Table S6 Summary of multiple alignments of tRNA genes in the mitogenomes of 12 Caliscelidae species.

Table S7 Nucleotide diversity (A) and the ratio of Ka/Ks (B) of PCGs from 16 reported Augilini mitogenomes.
