# Supplementary figures and images for: Comparative analysis of twelve mitogenomes of Caliscelidae (Hemiptera: Fulgoromorpha) and their phylogenetic implications
Source: PeerJ. 2021 Nov 16;9:e12465. doi: 10.7717/peerj.12465 (PMC8603831; doi:10.7717/peerj.12465)

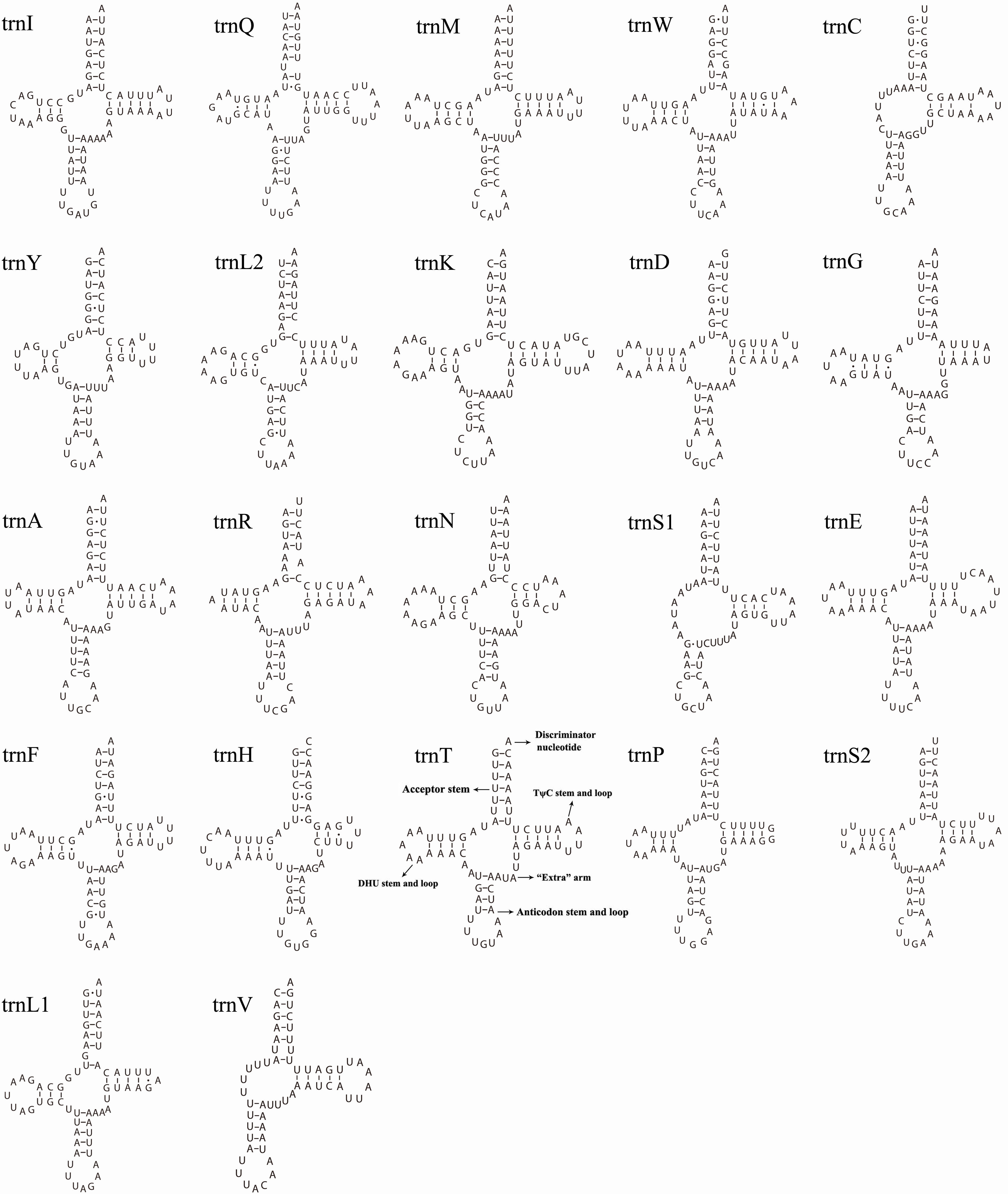

Supplement: Supplemental Information 1 — Figure S1. Secondary structures of the transfer RNAs in the mitogenome of Augilina tetraina. Dashes and dots indicate Watson–Crick and GU base pairing, respectively. Figure S2. Secondary structures of the transfer RNAs in the mitogenome of Augilina triaina. Dashes and dots indicate Watson–Crick and GU base pairing, respectively. Figure S3. Secondary structures of the transfer RNAs in the mitogenome of Symplana brevistrata. Dashes and dots indicate Watson–Crick and GU base pairing, respectively. Figure S4. Secondary structures of the transfer RNAs in the mitogenome of Symplana lii. Dashes and dots indicate Watson–Crick and GU base pairing, respectively. Figure S5. Secondary structures of the transfer RNAs in the mitogenome of Neosymplana vittatum. Dashes and dots indicate Watson–Crick and GU base pairing, respectively. Figure S6. Secondary structures of the transfer RNAs in the mitogenome of Pseudosymplanella nigrifasciata. Dashes and dots indicate Watson–Crick and GU base pairing, respectively. Figure S7. Secondary structures of the transfer RNAs in the mitogenome of Symplanella brevicephala. Dashes and dots indicate Watson–Crick and GU base pairing, respectively. Figure S8. Secondary structures of the transfer RNAs in the mitogenome of Symplanella unipuncta. Dashes and dots indicate Watson–Crick and GU base pairing, respectively. Figure S9. Secondary structures of the transfer RNAs in the mitogenome of Augilodes binghami. Dashes and dots indicate Watson–Crick and GU base pairing, respectively. Figure S10. Secondary structures of the transfer RNAs in the mitogenome of Cylindratus longicephalus. Dashes and dots indicate Watson–Crick and GU base pairing, respectively. Figure S11. Secondary structures of the transfer RNAs in the mitogenome of Caliscelis shandongensis. Dashes and dots indicate Watson–Crick and GU base pairing, respectively. Figure S12. Secondary structures of the transfer RNAs in the mitogenome of Peltonotellus sp. Dashes and dots indicate Watson–Crick [file peerj-09-12465-s001.zip › supplementary materials -figure and table/Figure S1.jpg]

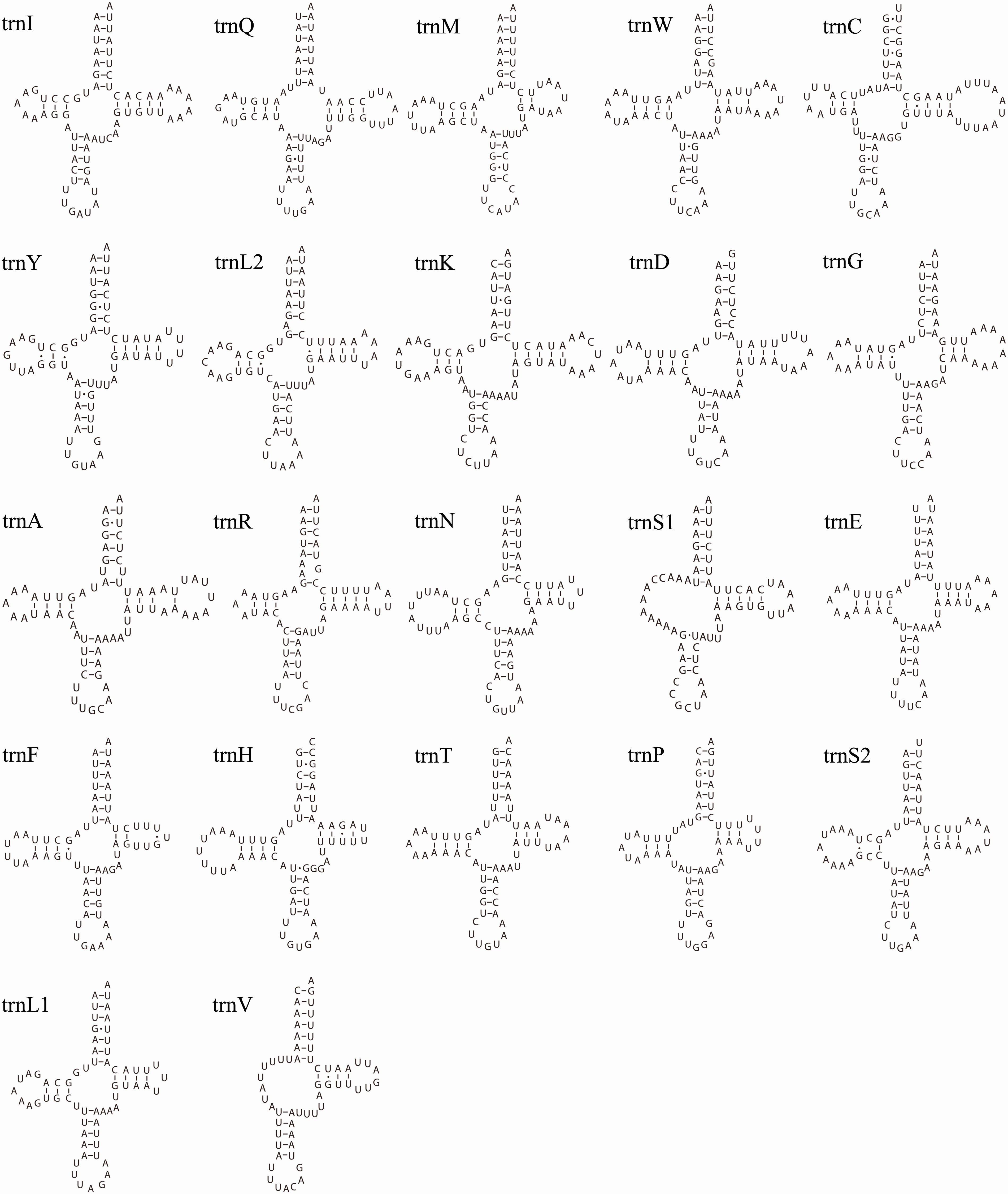

Supplement: Supplemental Information 1 — Figure S1. Secondary structures of the transfer RNAs in the mitogenome of Augilina tetraina. Dashes and dots indicate Watson–Crick and GU base pairing, respectively. Figure S2. Secondary structures of the transfer RNAs in the mitogenome of Augilina triaina. Dashes and dots indicate Watson–Crick and GU base pairing, respectively. Figure S3. Secondary structures of the transfer RNAs in the mitogenome of Symplana brevistrata. Dashes and dots indicate Watson–Crick and GU base pairing, respectively. Figure S4. Secondary structures of the transfer RNAs in the mitogenome of Symplana lii. Dashes and dots indicate Watson–Crick and GU base pairing, respectively. Figure S5. Secondary structures of the transfer RNAs in the mitogenome of Neosymplana vittatum. Dashes and dots indicate Watson–Crick and GU base pairing, respectively. Figure S6. Secondary structures of the transfer RNAs in the mitogenome of Pseudosymplanella nigrifasciata. Dashes and dots indicate Watson–Crick and GU base pairing, respectively. Figure S7. Secondary structures of the transfer RNAs in the mitogenome of Symplanella brevicephala. Dashes and dots indicate Watson–Crick and GU base pairing, respectively. Figure S8. Secondary structures of the transfer RNAs in the mitogenome of Symplanella unipuncta. Dashes and dots indicate Watson–Crick and GU base pairing, respectively. Figure S9. Secondary structures of the transfer RNAs in the mitogenome of Augilodes binghami. Dashes and dots indicate Watson–Crick and GU base pairing, respectively. Figure S10. Secondary structures of the transfer RNAs in the mitogenome of Cylindratus longicephalus. Dashes and dots indicate Watson–Crick and GU base pairing, respectively. Figure S11. Secondary structures of the transfer RNAs in the mitogenome of Caliscelis shandongensis. Dashes and dots indicate Watson–Crick and GU base pairing, respectively. Figure S12. Secondary structures of the transfer RNAs in the mitogenome of Peltonotellus sp. Dashes and dots indicate Watson–Crick [file peerj-09-12465-s001.zip › supplementary materials -figure and table/Figure S10.jpg]

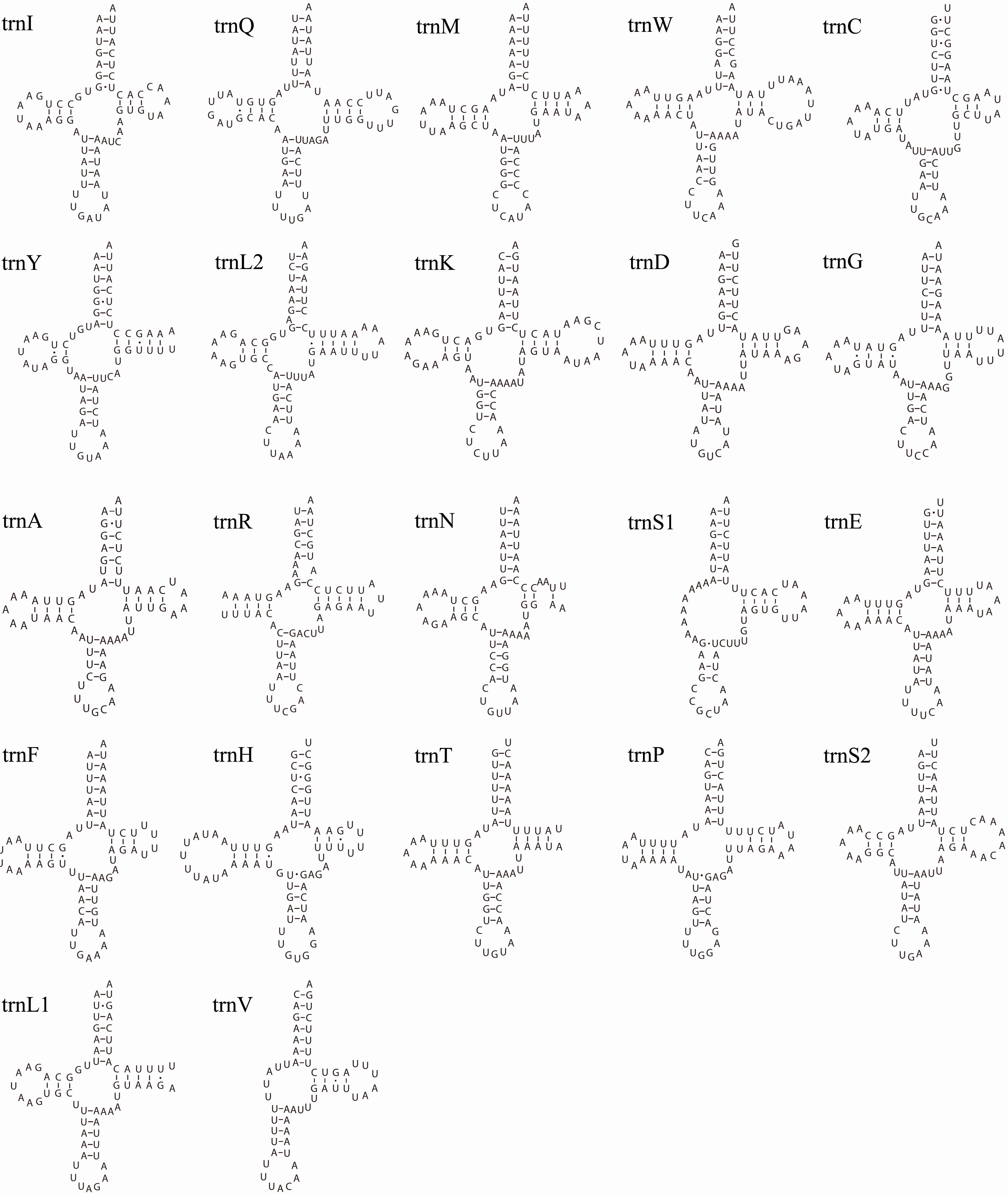

Supplement: Supplemental Information 1 — Figure S1. Secondary structures of the transfer RNAs in the mitogenome of Augilina tetraina. Dashes and dots indicate Watson–Crick and GU base pairing, respectively. Figure S2. Secondary structures of the transfer RNAs in the mitogenome of Augilina triaina. Dashes and dots indicate Watson–Crick and GU base pairing, respectively. Figure S3. Secondary structures of the transfer RNAs in the mitogenome of Symplana brevistrata. Dashes and dots indicate Watson–Crick and GU base pairing, respectively. Figure S4. Secondary structures of the transfer RNAs in the mitogenome of Symplana lii. Dashes and dots indicate Watson–Crick and GU base pairing, respectively. Figure S5. Secondary structures of the transfer RNAs in the mitogenome of Neosymplana vittatum. Dashes and dots indicate Watson–Crick and GU base pairing, respectively. Figure S6. Secondary structures of the transfer RNAs in the mitogenome of Pseudosymplanella nigrifasciata. Dashes and dots indicate Watson–Crick and GU base pairing, respectively. Figure S7. Secondary structures of the transfer RNAs in the mitogenome of Symplanella brevicephala. Dashes and dots indicate Watson–Crick and GU base pairing, respectively. Figure S8. Secondary structures of the transfer RNAs in the mitogenome of Symplanella unipuncta. Dashes and dots indicate Watson–Crick and GU base pairing, respectively. Figure S9. Secondary structures of the transfer RNAs in the mitogenome of Augilodes binghami. Dashes and dots indicate Watson–Crick and GU base pairing, respectively. Figure S10. Secondary structures of the transfer RNAs in the mitogenome of Cylindratus longicephalus. Dashes and dots indicate Watson–Crick and GU base pairing, respectively. Figure S11. Secondary structures of the transfer RNAs in the mitogenome of Caliscelis shandongensis. Dashes and dots indicate Watson–Crick and GU base pairing, respectively. Figure S12. Secondary structures of the transfer RNAs in the mitogenome of Peltonotellus sp. Dashes and dots indicate Watson–Crick [file peerj-09-12465-s001.zip › supplementary materials -figure and table/Figure S11.jpg]

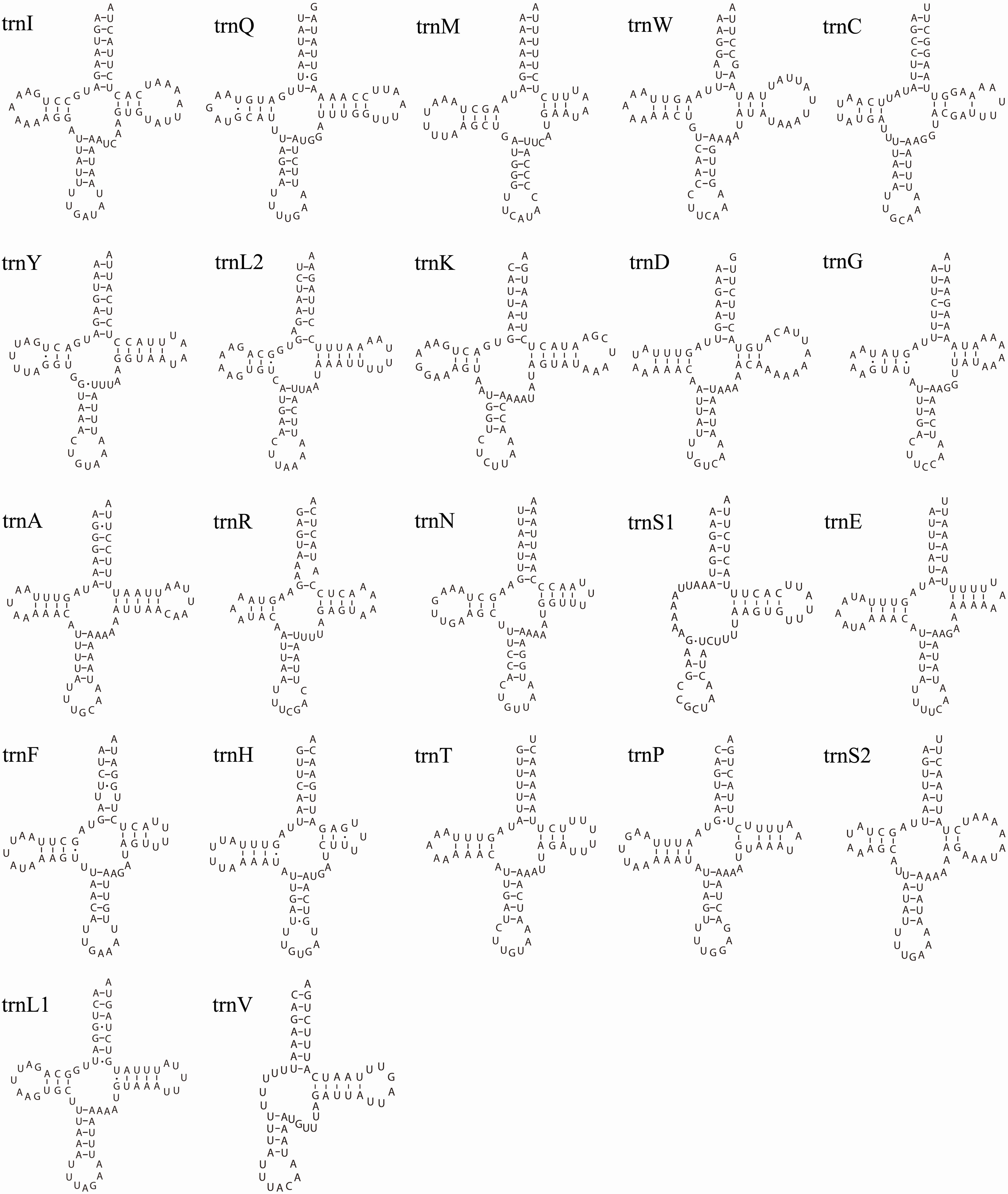

Supplement: Supplemental Information 1 — Figure S1. Secondary structures of the transfer RNAs in the mitogenome of Augilina tetraina. Dashes and dots indicate Watson–Crick and GU base pairing, respectively. Figure S2. Secondary structures of the transfer RNAs in the mitogenome of Augilina triaina. Dashes and dots indicate Watson–Crick and GU base pairing, respectively. Figure S3. Secondary structures of the transfer RNAs in the mitogenome of Symplana brevistrata. Dashes and dots indicate Watson–Crick and GU base pairing, respectively. Figure S4. Secondary structures of the transfer RNAs in the mitogenome of Symplana lii. Dashes and dots indicate Watson–Crick and GU base pairing, respectively. Figure S5. Secondary structures of the transfer RNAs in the mitogenome of Neosymplana vittatum. Dashes and dots indicate Watson–Crick and GU base pairing, respectively. Figure S6. Secondary structures of the transfer RNAs in the mitogenome of Pseudosymplanella nigrifasciata. Dashes and dots indicate Watson–Crick and GU base pairing, respectively. Figure S7. Secondary structures of the transfer RNAs in the mitogenome of Symplanella brevicephala. Dashes and dots indicate Watson–Crick and GU base pairing, respectively. Figure S8. Secondary structures of the transfer RNAs in the mitogenome of Symplanella unipuncta. Dashes and dots indicate Watson–Crick and GU base pairing, respectively. Figure S9. Secondary structures of the transfer RNAs in the mitogenome of Augilodes binghami. Dashes and dots indicate Watson–Crick and GU base pairing, respectively. Figure S10. Secondary structures of the transfer RNAs in the mitogenome of Cylindratus longicephalus. Dashes and dots indicate Watson–Crick and GU base pairing, respectively. Figure S11. Secondary structures of the transfer RNAs in the mitogenome of Caliscelis shandongensis. Dashes and dots indicate Watson–Crick and GU base pairing, respectively. Figure S12. Secondary structures of the transfer RNAs in the mitogenome of Peltonotellus sp. Dashes and dots indicate Watson–Crick [file peerj-09-12465-s001.zip › supplementary materials -figure and table/Figure S12.jpg]

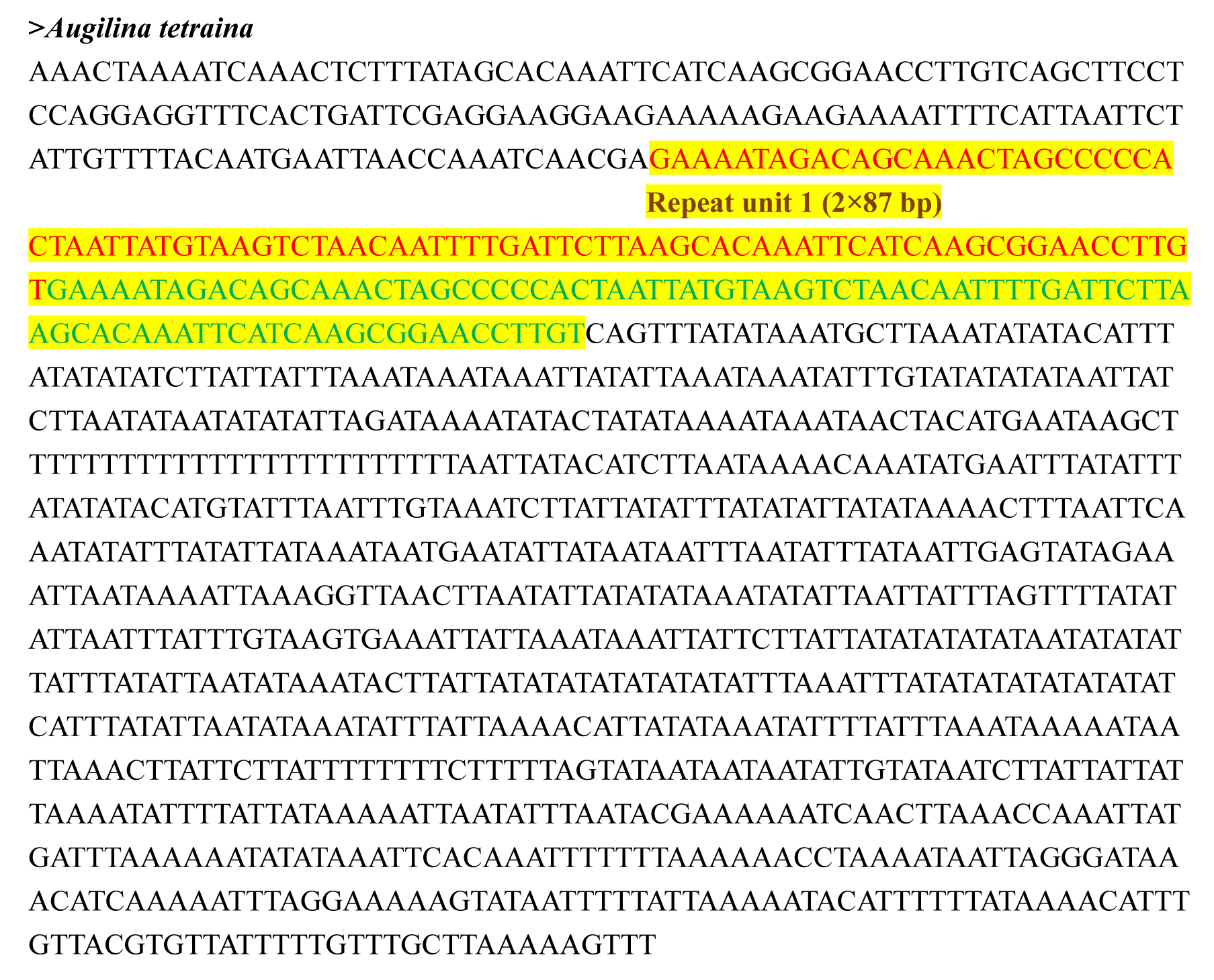

Supplement: Supplemental Information 1 — Figure S1. Secondary structures of the transfer RNAs in the mitogenome of Augilina tetraina. Dashes and dots indicate Watson–Crick and GU base pairing, respectively. Figure S2. Secondary structures of the transfer RNAs in the mitogenome of Augilina triaina. Dashes and dots indicate Watson–Crick and GU base pairing, respectively. Figure S3. Secondary structures of the transfer RNAs in the mitogenome of Symplana brevistrata. Dashes and dots indicate Watson–Crick and GU base pairing, respectively. Figure S4. Secondary structures of the transfer RNAs in the mitogenome of Symplana lii. Dashes and dots indicate Watson–Crick and GU base pairing, respectively. Figure S5. Secondary structures of the transfer RNAs in the mitogenome of Neosymplana vittatum. Dashes and dots indicate Watson–Crick and GU base pairing, respectively. Figure S6. Secondary structures of the transfer RNAs in the mitogenome of Pseudosymplanella nigrifasciata. Dashes and dots indicate Watson–Crick and GU base pairing, respectively. Figure S7. Secondary structures of the transfer RNAs in the mitogenome of Symplanella brevicephala. Dashes and dots indicate Watson–Crick and GU base pairing, respectively. Figure S8. Secondary structures of the transfer RNAs in the mitogenome of Symplanella unipuncta. Dashes and dots indicate Watson–Crick and GU base pairing, respectively. Figure S9. Secondary structures of the transfer RNAs in the mitogenome of Augilodes binghami. Dashes and dots indicate Watson–Crick and GU base pairing, respectively. Figure S10. Secondary structures of the transfer RNAs in the mitogenome of Cylindratus longicephalus. Dashes and dots indicate Watson–Crick and GU base pairing, respectively. Figure S11. Secondary structures of the transfer RNAs in the mitogenome of Caliscelis shandongensis. Dashes and dots indicate Watson–Crick and GU base pairing, respectively. Figure S12. Secondary structures of the transfer RNAs in the mitogenome of Peltonotellus sp. Dashes and dots indicate Watson–Crick [file peerj-09-12465-s001.zip › supplementary materials -figure and table/Figure S13.tif]

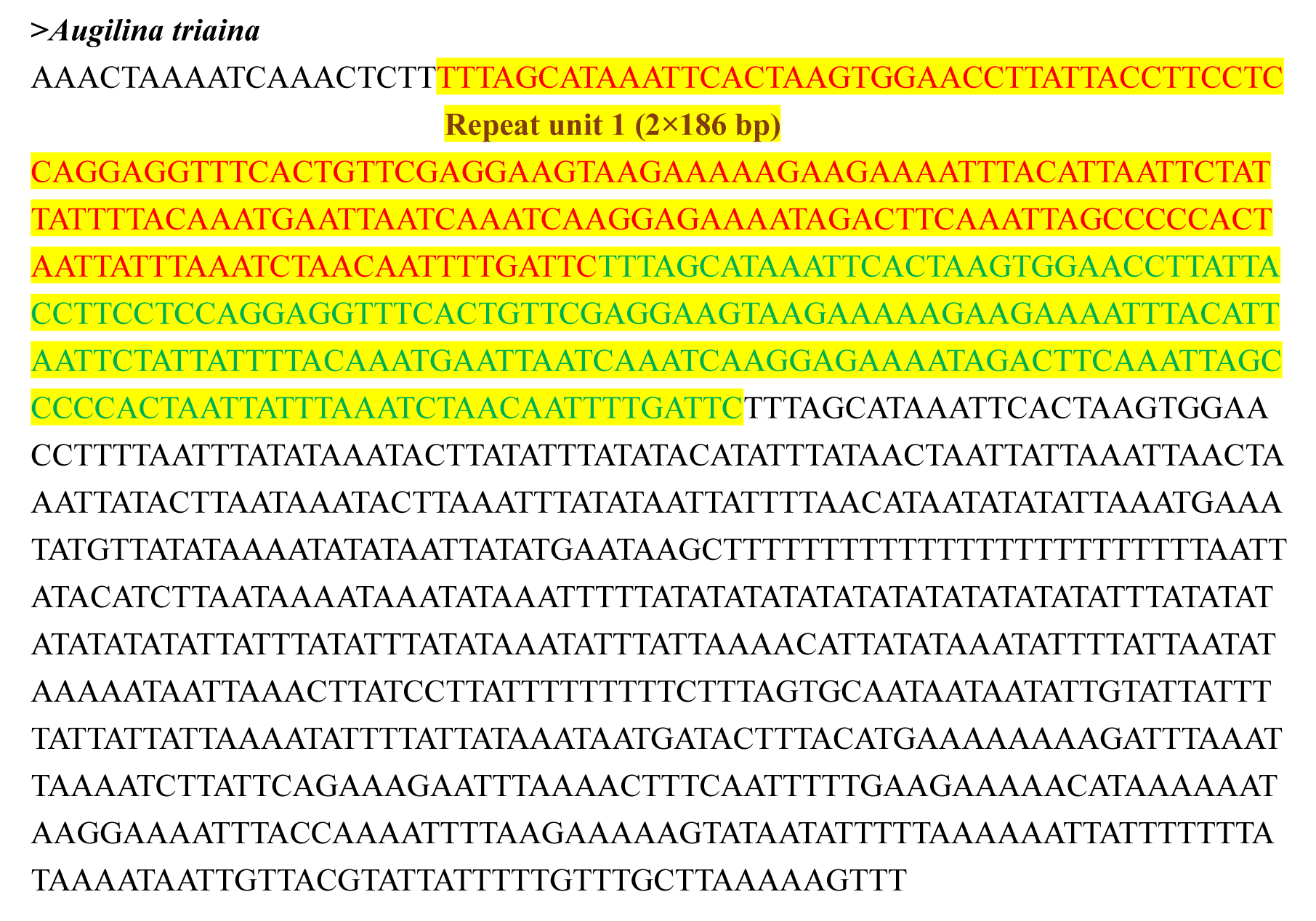

Supplement: Supplemental Information 1 — Figure S1. Secondary structures of the transfer RNAs in the mitogenome of Augilina tetraina. Dashes and dots indicate Watson–Crick and GU base pairing, respectively. Figure S2. Secondary structures of the transfer RNAs in the mitogenome of Augilina triaina. Dashes and dots indicate Watson–Crick and GU base pairing, respectively. Figure S3. Secondary structures of the transfer RNAs in the mitogenome of Symplana brevistrata. Dashes and dots indicate Watson–Crick and GU base pairing, respectively. Figure S4. Secondary structures of the transfer RNAs in the mitogenome of Symplana lii. Dashes and dots indicate Watson–Crick and GU base pairing, respectively. Figure S5. Secondary structures of the transfer RNAs in the mitogenome of Neosymplana vittatum. Dashes and dots indicate Watson–Crick and GU base pairing, respectively. Figure S6. Secondary structures of the transfer RNAs in the mitogenome of Pseudosymplanella nigrifasciata. Dashes and dots indicate Watson–Crick and GU base pairing, respectively. Figure S7. Secondary structures of the transfer RNAs in the mitogenome of Symplanella brevicephala. Dashes and dots indicate Watson–Crick and GU base pairing, respectively. Figure S8. Secondary structures of the transfer RNAs in the mitogenome of Symplanella unipuncta. Dashes and dots indicate Watson–Crick and GU base pairing, respectively. Figure S9. Secondary structures of the transfer RNAs in the mitogenome of Augilodes binghami. Dashes and dots indicate Watson–Crick and GU base pairing, respectively. Figure S10. Secondary structures of the transfer RNAs in the mitogenome of Cylindratus longicephalus. Dashes and dots indicate Watson–Crick and GU base pairing, respectively. Figure S11. Secondary structures of the transfer RNAs in the mitogenome of Caliscelis shandongensis. Dashes and dots indicate Watson–Crick and GU base pairing, respectively. Figure S12. Secondary structures of the transfer RNAs in the mitogenome of Peltonotellus sp. Dashes and dots indicate Watson–Crick [file peerj-09-12465-s001.zip › supplementary materials -figure and table/Figure S14.tif]

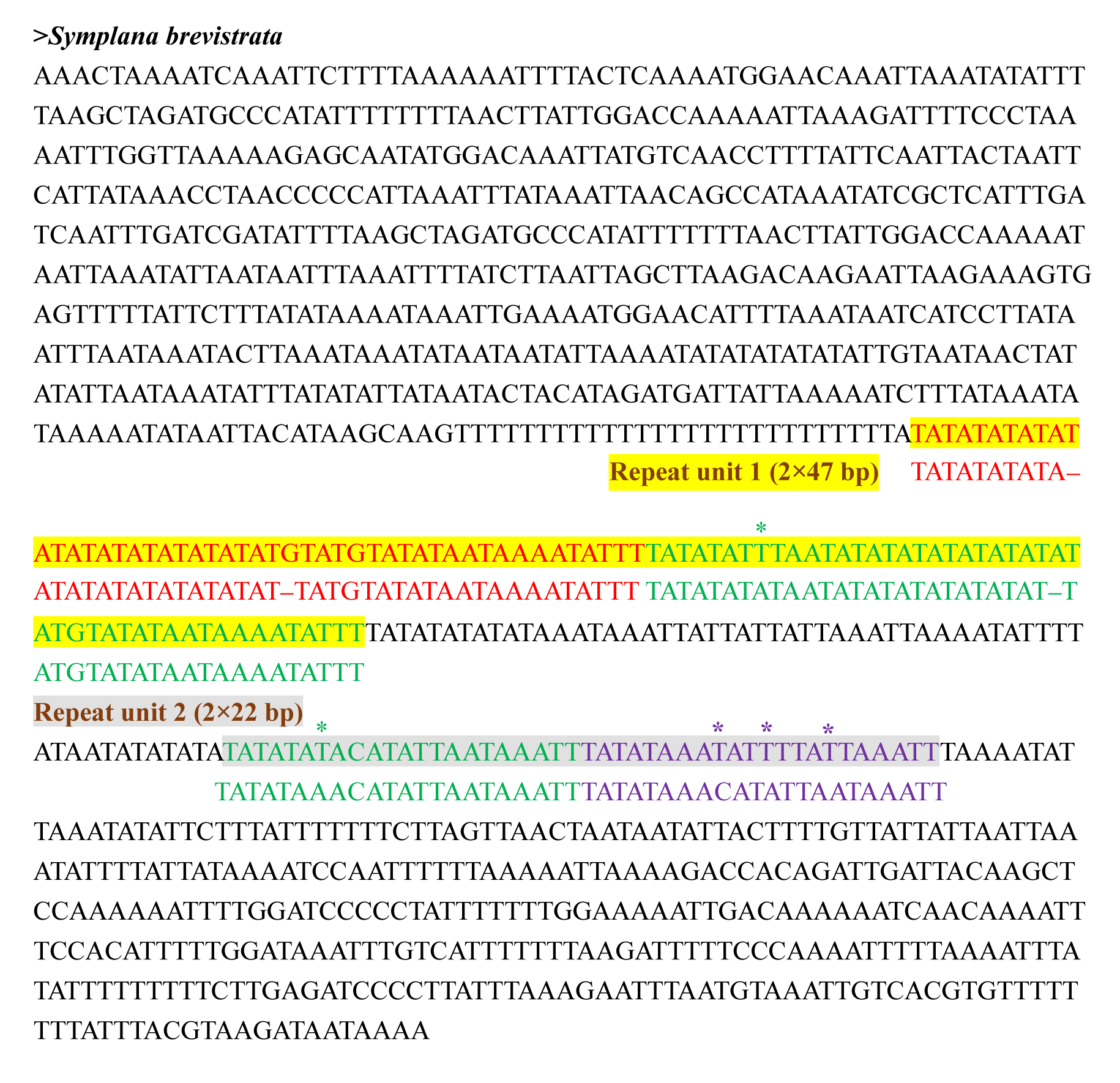

Supplement: Supplemental Information 1 — Figure S1. Secondary structures of the transfer RNAs in the mitogenome of Augilina tetraina. Dashes and dots indicate Watson–Crick and GU base pairing, respectively. Figure S2. Secondary structures of the transfer RNAs in the mitogenome of Augilina triaina. Dashes and dots indicate Watson–Crick and GU base pairing, respectively. Figure S3. Secondary structures of the transfer RNAs in the mitogenome of Symplana brevistrata. Dashes and dots indicate Watson–Crick and GU base pairing, respectively. Figure S4. Secondary structures of the transfer RNAs in the mitogenome of Symplana lii. Dashes and dots indicate Watson–Crick and GU base pairing, respectively. Figure S5. Secondary structures of the transfer RNAs in the mitogenome of Neosymplana vittatum. Dashes and dots indicate Watson–Crick and GU base pairing, respectively. Figure S6. Secondary structures of the transfer RNAs in the mitogenome of Pseudosymplanella nigrifasciata. Dashes and dots indicate Watson–Crick and GU base pairing, respectively. Figure S7. Secondary structures of the transfer RNAs in the mitogenome of Symplanella brevicephala. Dashes and dots indicate Watson–Crick and GU base pairing, respectively. Figure S8. Secondary structures of the transfer RNAs in the mitogenome of Symplanella unipuncta. Dashes and dots indicate Watson–Crick and GU base pairing, respectively. Figure S9. Secondary structures of the transfer RNAs in the mitogenome of Augilodes binghami. Dashes and dots indicate Watson–Crick and GU base pairing, respectively. Figure S10. Secondary structures of the transfer RNAs in the mitogenome of Cylindratus longicephalus. Dashes and dots indicate Watson–Crick and GU base pairing, respectively. Figure S11. Secondary structures of the transfer RNAs in the mitogenome of Caliscelis shandongensis. Dashes and dots indicate Watson–Crick and GU base pairing, respectively. Figure S12. Secondary structures of the transfer RNAs in the mitogenome of Peltonotellus sp. Dashes and dots indicate Watson–Crick [file peerj-09-12465-s001.zip › supplementary materials -figure and table/Figure S15.tif]

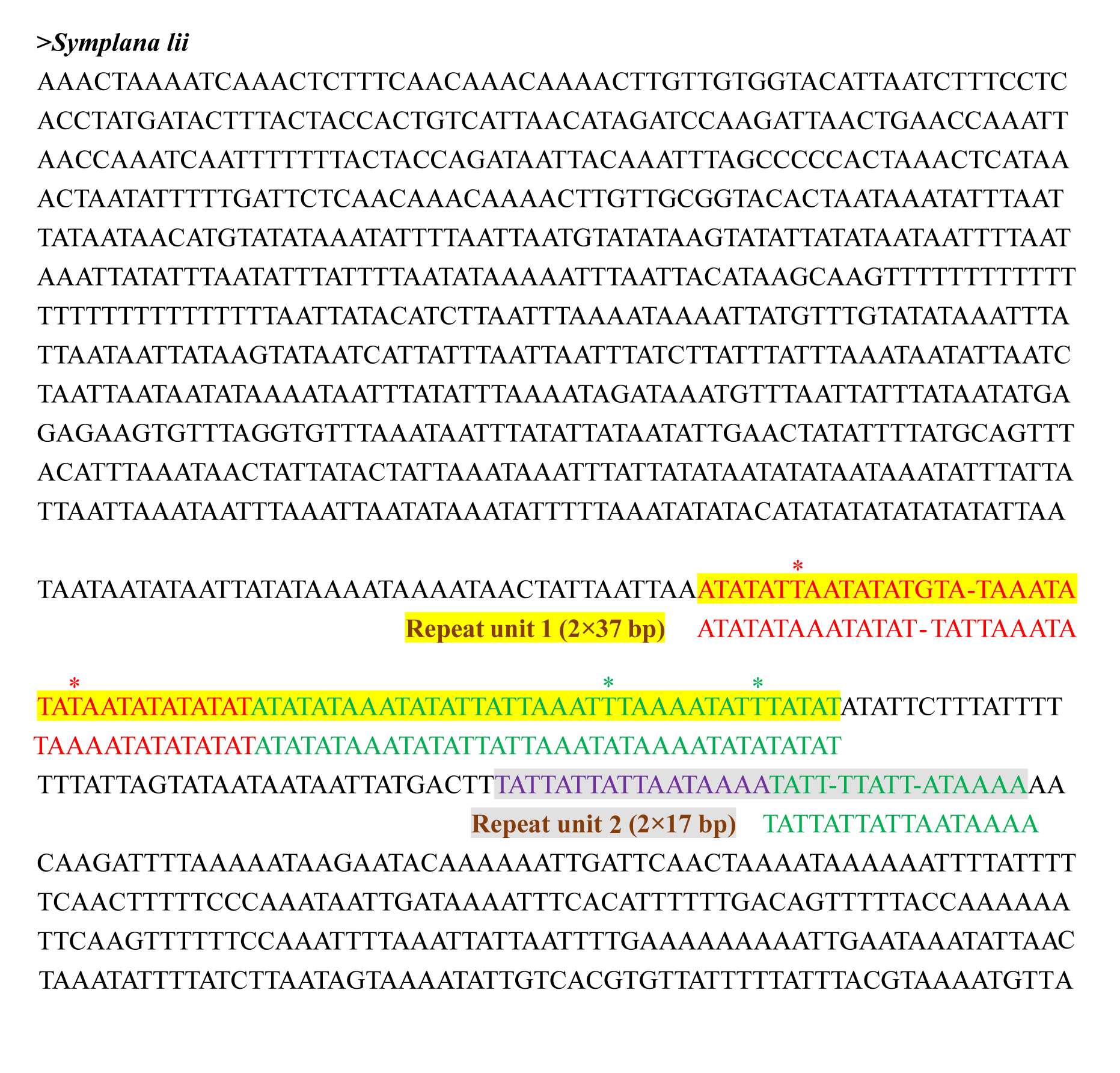

Supplement: Supplemental Information 1 — Figure S1. Secondary structures of the transfer RNAs in the mitogenome of Augilina tetraina. Dashes and dots indicate Watson–Crick and GU base pairing, respectively. Figure S2. Secondary structures of the transfer RNAs in the mitogenome of Augilina triaina. Dashes and dots indicate Watson–Crick and GU base pairing, respectively. Figure S3. Secondary structures of the transfer RNAs in the mitogenome of Symplana brevistrata. Dashes and dots indicate Watson–Crick and GU base pairing, respectively. Figure S4. Secondary structures of the transfer RNAs in the mitogenome of Symplana lii. Dashes and dots indicate Watson–Crick and GU base pairing, respectively. Figure S5. Secondary structures of the transfer RNAs in the mitogenome of Neosymplana vittatum. Dashes and dots indicate Watson–Crick and GU base pairing, respectively. Figure S6. Secondary structures of the transfer RNAs in the mitogenome of Pseudosymplanella nigrifasciata. Dashes and dots indicate Watson–Crick and GU base pairing, respectively. Figure S7. Secondary structures of the transfer RNAs in the mitogenome of Symplanella brevicephala. Dashes and dots indicate Watson–Crick and GU base pairing, respectively. Figure S8. Secondary structures of the transfer RNAs in the mitogenome of Symplanella unipuncta. Dashes and dots indicate Watson–Crick and GU base pairing, respectively. Figure S9. Secondary structures of the transfer RNAs in the mitogenome of Augilodes binghami. Dashes and dots indicate Watson–Crick and GU base pairing, respectively. Figure S10. Secondary structures of the transfer RNAs in the mitogenome of Cylindratus longicephalus. Dashes and dots indicate Watson–Crick and GU base pairing, respectively. Figure S11. Secondary structures of the transfer RNAs in the mitogenome of Caliscelis shandongensis. Dashes and dots indicate Watson–Crick and GU base pairing, respectively. Figure S12. Secondary structures of the transfer RNAs in the mitogenome of Peltonotellus sp. Dashes and dots indicate Watson–Crick [file peerj-09-12465-s001.zip › supplementary materials -figure and table/Figure S16.tif]

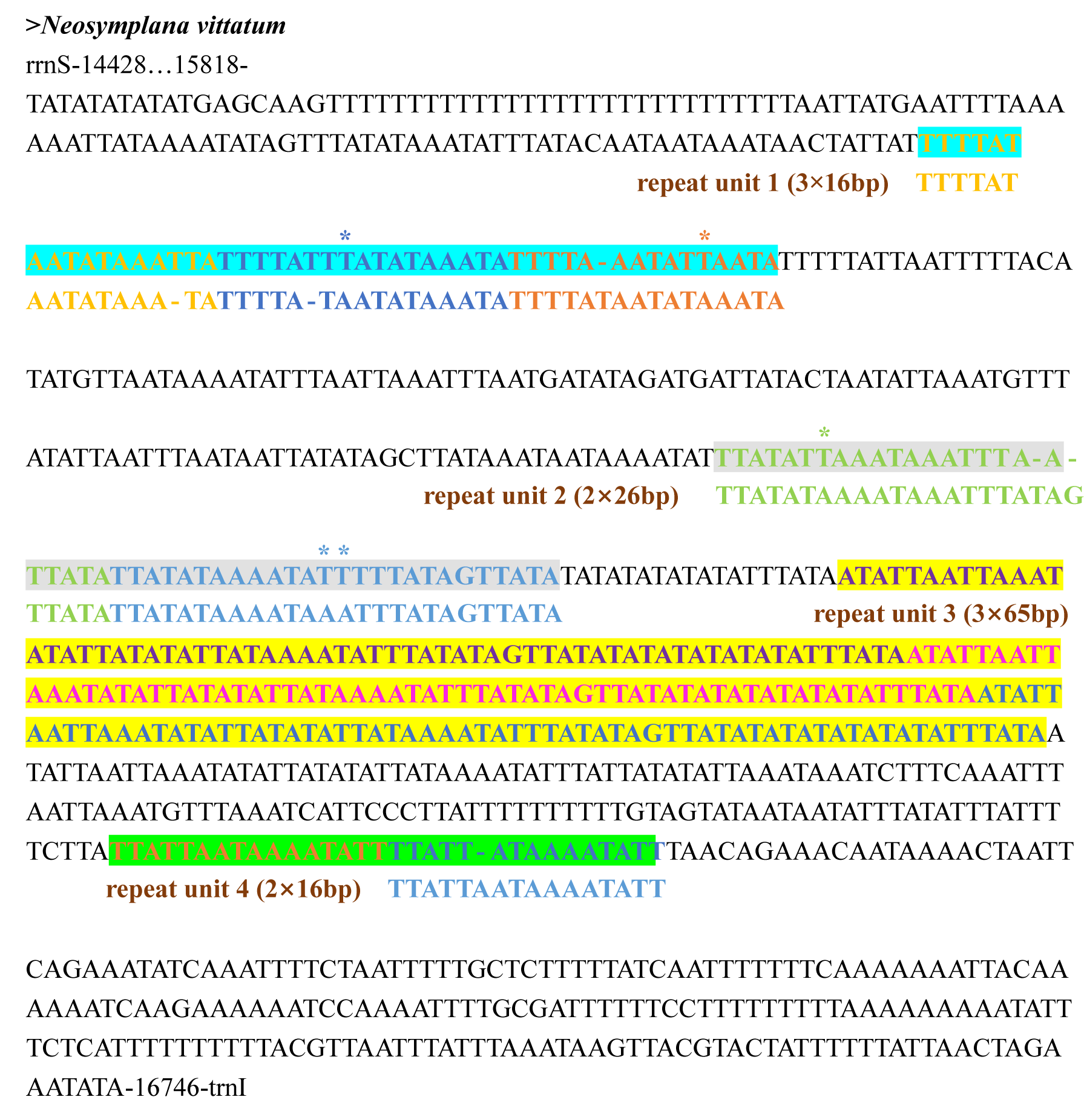

Supplement: Supplemental Information 1 — Figure S1. Secondary structures of the transfer RNAs in the mitogenome of Augilina tetraina. Dashes and dots indicate Watson–Crick and GU base pairing, respectively. Figure S2. Secondary structures of the transfer RNAs in the mitogenome of Augilina triaina. Dashes and dots indicate Watson–Crick and GU base pairing, respectively. Figure S3. Secondary structures of the transfer RNAs in the mitogenome of Symplana brevistrata. Dashes and dots indicate Watson–Crick and GU base pairing, respectively. Figure S4. Secondary structures of the transfer RNAs in the mitogenome of Symplana lii. Dashes and dots indicate Watson–Crick and GU base pairing, respectively. Figure S5. Secondary structures of the transfer RNAs in the mitogenome of Neosymplana vittatum. Dashes and dots indicate Watson–Crick and GU base pairing, respectively. Figure S6. Secondary structures of the transfer RNAs in the mitogenome of Pseudosymplanella nigrifasciata. Dashes and dots indicate Watson–Crick and GU base pairing, respectively. Figure S7. Secondary structures of the transfer RNAs in the mitogenome of Symplanella brevicephala. Dashes and dots indicate Watson–Crick and GU base pairing, respectively. Figure S8. Secondary structures of the transfer RNAs in the mitogenome of Symplanella unipuncta. Dashes and dots indicate Watson–Crick and GU base pairing, respectively. Figure S9. Secondary structures of the transfer RNAs in the mitogenome of Augilodes binghami. Dashes and dots indicate Watson–Crick and GU base pairing, respectively. Figure S10. Secondary structures of the transfer RNAs in the mitogenome of Cylindratus longicephalus. Dashes and dots indicate Watson–Crick and GU base pairing, respectively. Figure S11. Secondary structures of the transfer RNAs in the mitogenome of Caliscelis shandongensis. Dashes and dots indicate Watson–Crick and GU base pairing, respectively. Figure S12. Secondary structures of the transfer RNAs in the mitogenome of Peltonotellus sp. Dashes and dots indicate Watson–Crick [file peerj-09-12465-s001.zip › supplementary materials -figure and table/Figure S17.tif]

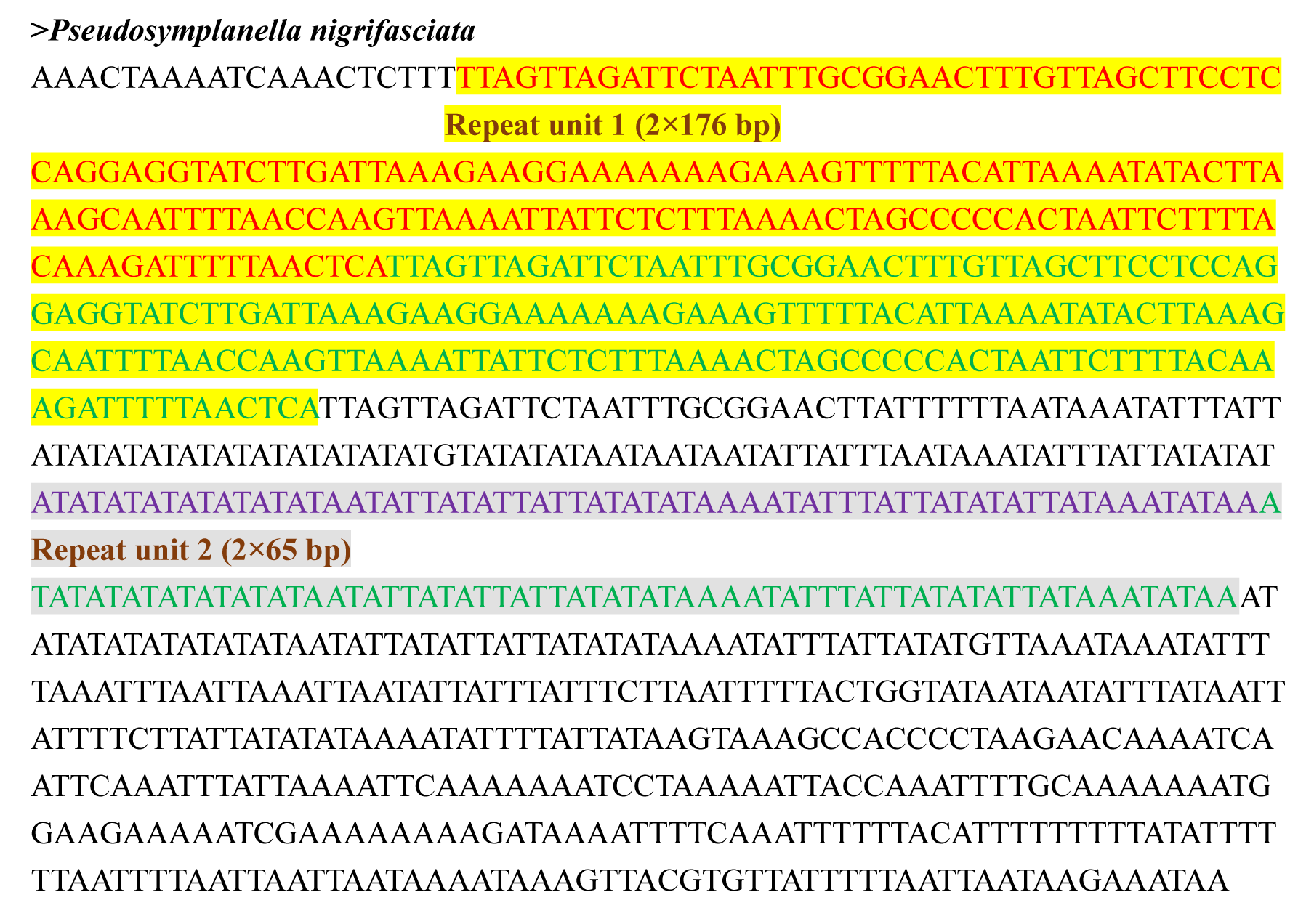

Supplement: Supplemental Information 1 — Figure S1. Secondary structures of the transfer RNAs in the mitogenome of Augilina tetraina. Dashes and dots indicate Watson–Crick and GU base pairing, respectively. Figure S2. Secondary structures of the transfer RNAs in the mitogenome of Augilina triaina. Dashes and dots indicate Watson–Crick and GU base pairing, respectively. Figure S3. Secondary structures of the transfer RNAs in the mitogenome of Symplana brevistrata. Dashes and dots indicate Watson–Crick and GU base pairing, respectively. Figure S4. Secondary structures of the transfer RNAs in the mitogenome of Symplana lii. Dashes and dots indicate Watson–Crick and GU base pairing, respectively. Figure S5. Secondary structures of the transfer RNAs in the mitogenome of Neosymplana vittatum. Dashes and dots indicate Watson–Crick and GU base pairing, respectively. Figure S6. Secondary structures of the transfer RNAs in the mitogenome of Pseudosymplanella nigrifasciata. Dashes and dots indicate Watson–Crick and GU base pairing, respectively. Figure S7. Secondary structures of the transfer RNAs in the mitogenome of Symplanella brevicephala. Dashes and dots indicate Watson–Crick and GU base pairing, respectively. Figure S8. Secondary structures of the transfer RNAs in the mitogenome of Symplanella unipuncta. Dashes and dots indicate Watson–Crick and GU base pairing, respectively. Figure S9. Secondary structures of the transfer RNAs in the mitogenome of Augilodes binghami. Dashes and dots indicate Watson–Crick and GU base pairing, respectively. Figure S10. Secondary structures of the transfer RNAs in the mitogenome of Cylindratus longicephalus. Dashes and dots indicate Watson–Crick and GU base pairing, respectively. Figure S11. Secondary structures of the transfer RNAs in the mitogenome of Caliscelis shandongensis. Dashes and dots indicate Watson–Crick and GU base pairing, respectively. Figure S12. Secondary structures of the transfer RNAs in the mitogenome of Peltonotellus sp. Dashes and dots indicate Watson–Crick [file peerj-09-12465-s001.zip › supplementary materials -figure and table/Figure S18.tif]

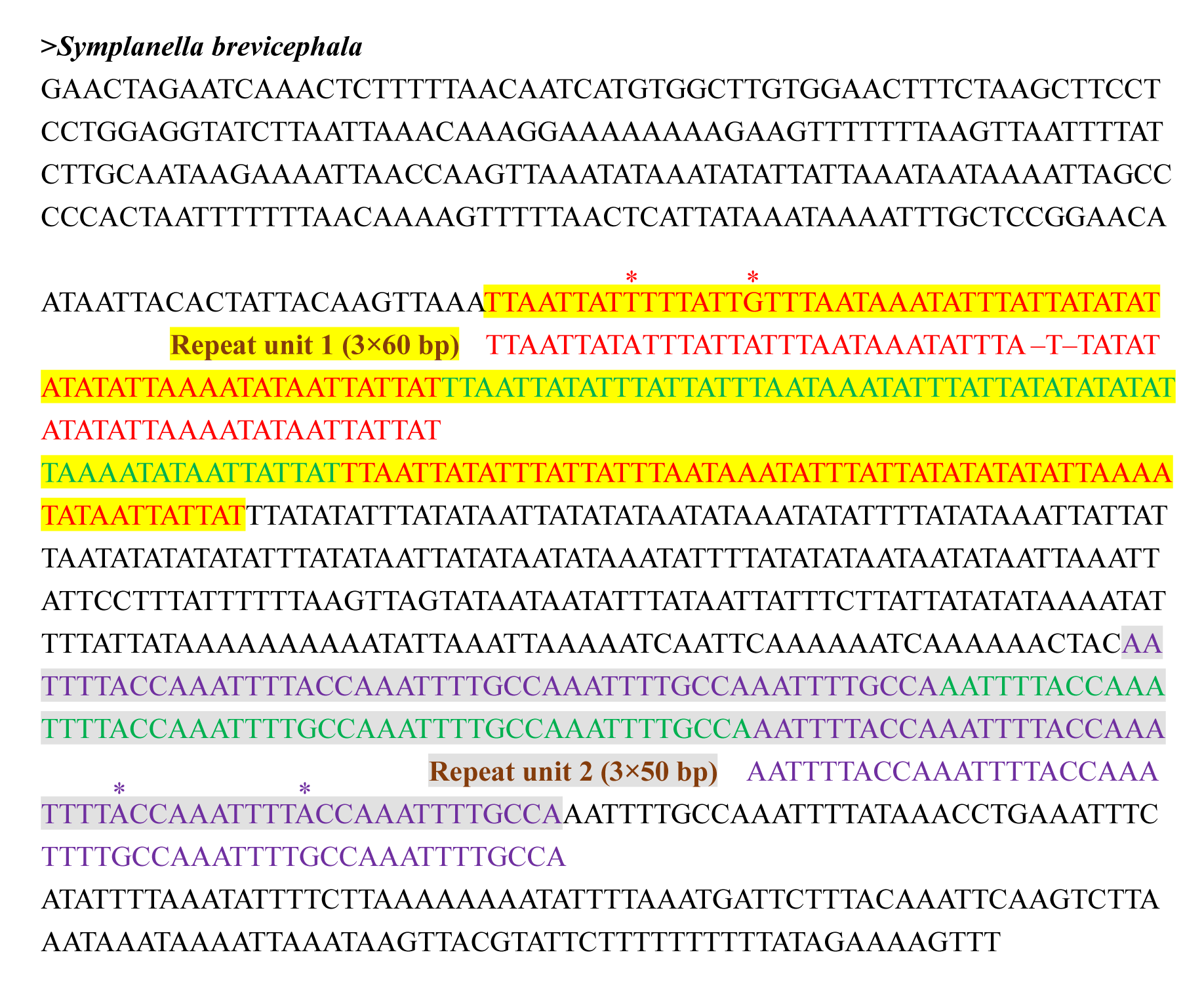

Supplement: Supplemental Information 1 — Figure S1. Secondary structures of the transfer RNAs in the mitogenome of Augilina tetraina. Dashes and dots indicate Watson–Crick and GU base pairing, respectively. Figure S2. Secondary structures of the transfer RNAs in the mitogenome of Augilina triaina. Dashes and dots indicate Watson–Crick and GU base pairing, respectively. Figure S3. Secondary structures of the transfer RNAs in the mitogenome of Symplana brevistrata. Dashes and dots indicate Watson–Crick and GU base pairing, respectively. Figure S4. Secondary structures of the transfer RNAs in the mitogenome of Symplana lii. Dashes and dots indicate Watson–Crick and GU base pairing, respectively. Figure S5. Secondary structures of the transfer RNAs in the mitogenome of Neosymplana vittatum. Dashes and dots indicate Watson–Crick and GU base pairing, respectively. Figure S6. Secondary structures of the transfer RNAs in the mitogenome of Pseudosymplanella nigrifasciata. Dashes and dots indicate Watson–Crick and GU base pairing, respectively. Figure S7. Secondary structures of the transfer RNAs in the mitogenome of Symplanella brevicephala. Dashes and dots indicate Watson–Crick and GU base pairing, respectively. Figure S8. Secondary structures of the transfer RNAs in the mitogenome of Symplanella unipuncta. Dashes and dots indicate Watson–Crick and GU base pairing, respectively. Figure S9. Secondary structures of the transfer RNAs in the mitogenome of Augilodes binghami. Dashes and dots indicate Watson–Crick and GU base pairing, respectively. Figure S10. Secondary structures of the transfer RNAs in the mitogenome of Cylindratus longicephalus. Dashes and dots indicate Watson–Crick and GU base pairing, respectively. Figure S11. Secondary structures of the transfer RNAs in the mitogenome of Caliscelis shandongensis. Dashes and dots indicate Watson–Crick and GU base pairing, respectively. Figure S12. Secondary structures of the transfer RNAs in the mitogenome of Peltonotellus sp. Dashes and dots indicate Watson–Crick [file peerj-09-12465-s001.zip › supplementary materials -figure and table/Figure S19.tif]

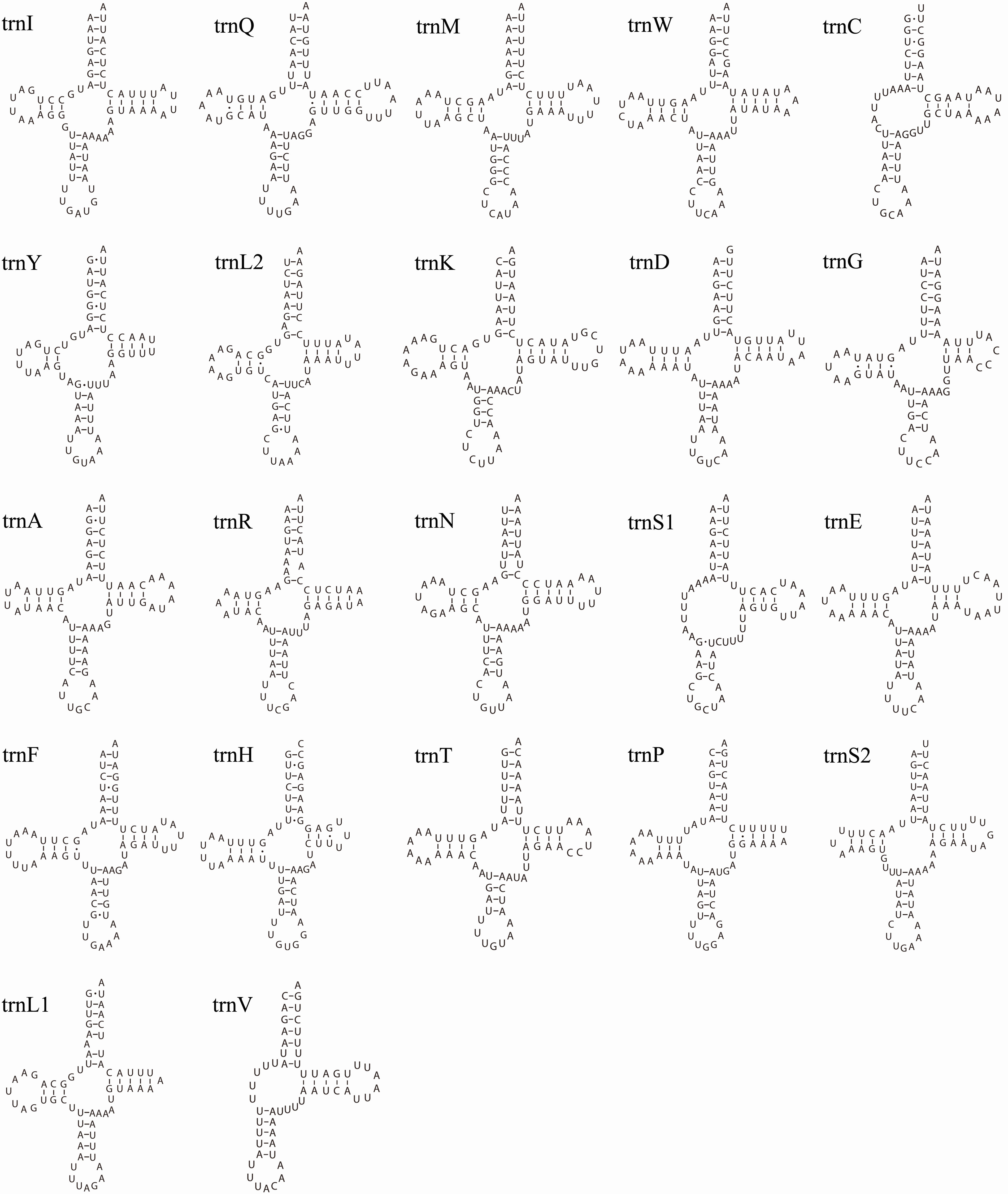

Supplement: Supplemental Information 1 — Figure S1. Secondary structures of the transfer RNAs in the mitogenome of Augilina tetraina. Dashes and dots indicate Watson–Crick and GU base pairing, respectively. Figure S2. Secondary structures of the transfer RNAs in the mitogenome of Augilina triaina. Dashes and dots indicate Watson–Crick and GU base pairing, respectively. Figure S3. Secondary structures of the transfer RNAs in the mitogenome of Symplana brevistrata. Dashes and dots indicate Watson–Crick and GU base pairing, respectively. Figure S4. Secondary structures of the transfer RNAs in the mitogenome of Symplana lii. Dashes and dots indicate Watson–Crick and GU base pairing, respectively. Figure S5. Secondary structures of the transfer RNAs in the mitogenome of Neosymplana vittatum. Dashes and dots indicate Watson–Crick and GU base pairing, respectively. Figure S6. Secondary structures of the transfer RNAs in the mitogenome of Pseudosymplanella nigrifasciata. Dashes and dots indicate Watson–Crick and GU base pairing, respectively. Figure S7. Secondary structures of the transfer RNAs in the mitogenome of Symplanella brevicephala. Dashes and dots indicate Watson–Crick and GU base pairing, respectively. Figure S8. Secondary structures of the transfer RNAs in the mitogenome of Symplanella unipuncta. Dashes and dots indicate Watson–Crick and GU base pairing, respectively. Figure S9. Secondary structures of the transfer RNAs in the mitogenome of Augilodes binghami. Dashes and dots indicate Watson–Crick and GU base pairing, respectively. Figure S10. Secondary structures of the transfer RNAs in the mitogenome of Cylindratus longicephalus. Dashes and dots indicate Watson–Crick and GU base pairing, respectively. Figure S11. Secondary structures of the transfer RNAs in the mitogenome of Caliscelis shandongensis. Dashes and dots indicate Watson–Crick and GU base pairing, respectively. Figure S12. Secondary structures of the transfer RNAs in the mitogenome of Peltonotellus sp. Dashes and dots indicate Watson–Crick [file peerj-09-12465-s001.zip › supplementary materials -figure and table/Figure S2.jpg]

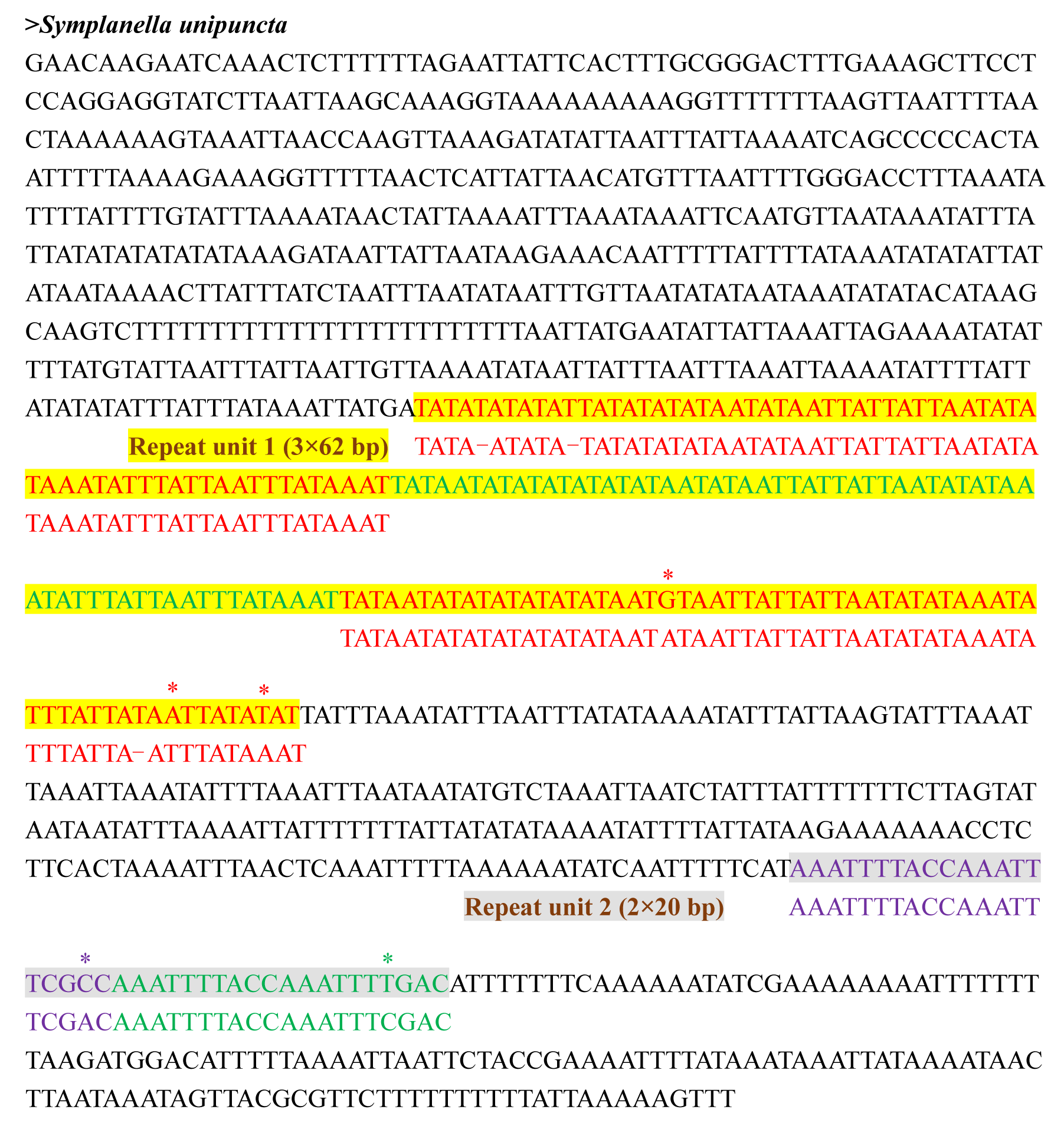

Supplement: Supplemental Information 1 — Figure S1. Secondary structures of the transfer RNAs in the mitogenome of Augilina tetraina. Dashes and dots indicate Watson–Crick and GU base pairing, respectively. Figure S2. Secondary structures of the transfer RNAs in the mitogenome of Augilina triaina. Dashes and dots indicate Watson–Crick and GU base pairing, respectively. Figure S3. Secondary structures of the transfer RNAs in the mitogenome of Symplana brevistrata. Dashes and dots indicate Watson–Crick and GU base pairing, respectively. Figure S4. Secondary structures of the transfer RNAs in the mitogenome of Symplana lii. Dashes and dots indicate Watson–Crick and GU base pairing, respectively. Figure S5. Secondary structures of the transfer RNAs in the mitogenome of Neosymplana vittatum. Dashes and dots indicate Watson–Crick and GU base pairing, respectively. Figure S6. Secondary structures of the transfer RNAs in the mitogenome of Pseudosymplanella nigrifasciata. Dashes and dots indicate Watson–Crick and GU base pairing, respectively. Figure S7. Secondary structures of the transfer RNAs in the mitogenome of Symplanella brevicephala. Dashes and dots indicate Watson–Crick and GU base pairing, respectively. Figure S8. Secondary structures of the transfer RNAs in the mitogenome of Symplanella unipuncta. Dashes and dots indicate Watson–Crick and GU base pairing, respectively. Figure S9. Secondary structures of the transfer RNAs in the mitogenome of Augilodes binghami. Dashes and dots indicate Watson–Crick and GU base pairing, respectively. Figure S10. Secondary structures of the transfer RNAs in the mitogenome of Cylindratus longicephalus. Dashes and dots indicate Watson–Crick and GU base pairing, respectively. Figure S11. Secondary structures of the transfer RNAs in the mitogenome of Caliscelis shandongensis. Dashes and dots indicate Watson–Crick and GU base pairing, respectively. Figure S12. Secondary structures of the transfer RNAs in the mitogenome of Peltonotellus sp. Dashes and dots indicate Watson–Crick [file peerj-09-12465-s001.zip › supplementary materials -figure and table/Figure S20.tif]

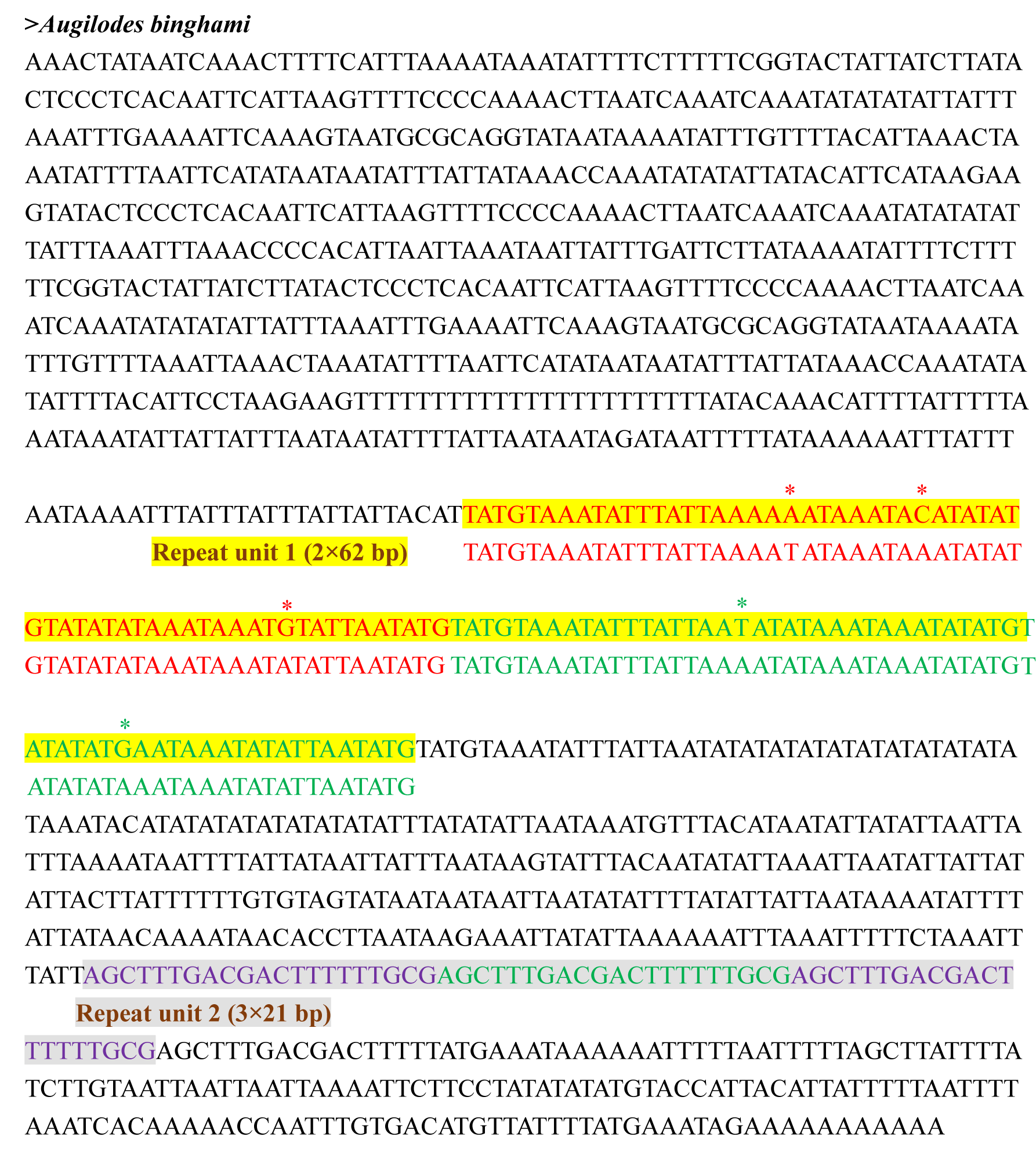

Supplement: Supplemental Information 1 — Figure S1. Secondary structures of the transfer RNAs in the mitogenome of Augilina tetraina. Dashes and dots indicate Watson–Crick and GU base pairing, respectively. Figure S2. Secondary structures of the transfer RNAs in the mitogenome of Augilina triaina. Dashes and dots indicate Watson–Crick and GU base pairing, respectively. Figure S3. Secondary structures of the transfer RNAs in the mitogenome of Symplana brevistrata. Dashes and dots indicate Watson–Crick and GU base pairing, respectively. Figure S4. Secondary structures of the transfer RNAs in the mitogenome of Symplana lii. Dashes and dots indicate Watson–Crick and GU base pairing, respectively. Figure S5. Secondary structures of the transfer RNAs in the mitogenome of Neosymplana vittatum. Dashes and dots indicate Watson–Crick and GU base pairing, respectively. Figure S6. Secondary structures of the transfer RNAs in the mitogenome of Pseudosymplanella nigrifasciata. Dashes and dots indicate Watson–Crick and GU base pairing, respectively. Figure S7. Secondary structures of the transfer RNAs in the mitogenome of Symplanella brevicephala. Dashes and dots indicate Watson–Crick and GU base pairing, respectively. Figure S8. Secondary structures of the transfer RNAs in the mitogenome of Symplanella unipuncta. Dashes and dots indicate Watson–Crick and GU base pairing, respectively. Figure S9. Secondary structures of the transfer RNAs in the mitogenome of Augilodes binghami. Dashes and dots indicate Watson–Crick and GU base pairing, respectively. Figure S10. Secondary structures of the transfer RNAs in the mitogenome of Cylindratus longicephalus. Dashes and dots indicate Watson–Crick and GU base pairing, respectively. Figure S11. Secondary structures of the transfer RNAs in the mitogenome of Caliscelis shandongensis. Dashes and dots indicate Watson–Crick and GU base pairing, respectively. Figure S12. Secondary structures of the transfer RNAs in the mitogenome of Peltonotellus sp. Dashes and dots indicate Watson–Crick [file peerj-09-12465-s001.zip › supplementary materials -figure and table/Figure S21.tif]

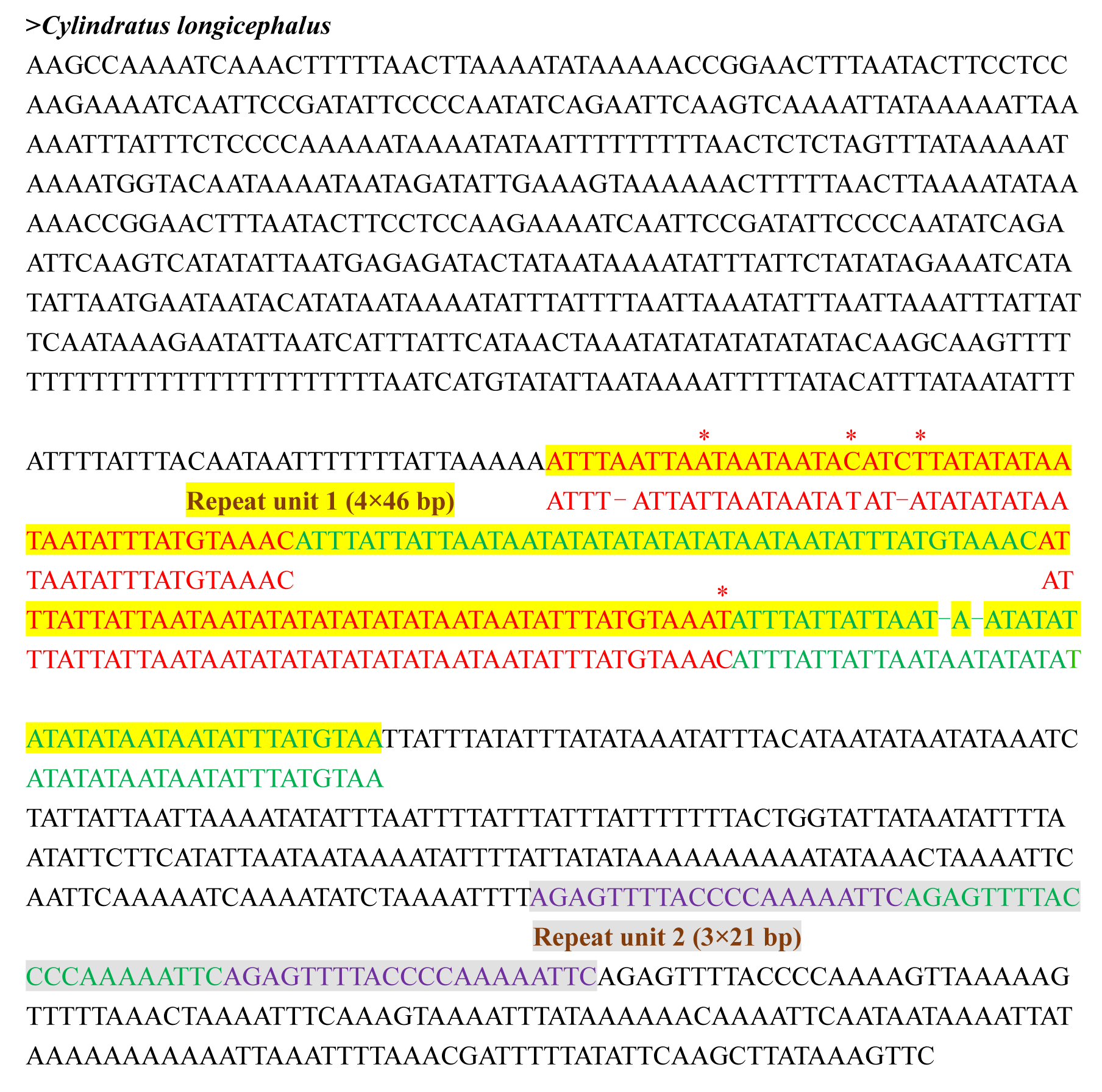

Supplement: Supplemental Information 1 — Figure S1. Secondary structures of the transfer RNAs in the mitogenome of Augilina tetraina. Dashes and dots indicate Watson–Crick and GU base pairing, respectively. Figure S2. Secondary structures of the transfer RNAs in the mitogenome of Augilina triaina. Dashes and dots indicate Watson–Crick and GU base pairing, respectively. Figure S3. Secondary structures of the transfer RNAs in the mitogenome of Symplana brevistrata. Dashes and dots indicate Watson–Crick and GU base pairing, respectively. Figure S4. Secondary structures of the transfer RNAs in the mitogenome of Symplana lii. Dashes and dots indicate Watson–Crick and GU base pairing, respectively. Figure S5. Secondary structures of the transfer RNAs in the mitogenome of Neosymplana vittatum. Dashes and dots indicate Watson–Crick and GU base pairing, respectively. Figure S6. Secondary structures of the transfer RNAs in the mitogenome of Pseudosymplanella nigrifasciata. Dashes and dots indicate Watson–Crick and GU base pairing, respectively. Figure S7. Secondary structures of the transfer RNAs in the mitogenome of Symplanella brevicephala. Dashes and dots indicate Watson–Crick and GU base pairing, respectively. Figure S8. Secondary structures of the transfer RNAs in the mitogenome of Symplanella unipuncta. Dashes and dots indicate Watson–Crick and GU base pairing, respectively. Figure S9. Secondary structures of the transfer RNAs in the mitogenome of Augilodes binghami. Dashes and dots indicate Watson–Crick and GU base pairing, respectively. Figure S10. Secondary structures of the transfer RNAs in the mitogenome of Cylindratus longicephalus. Dashes and dots indicate Watson–Crick and GU base pairing, respectively. Figure S11. Secondary structures of the transfer RNAs in the mitogenome of Caliscelis shandongensis. Dashes and dots indicate Watson–Crick and GU base pairing, respectively. Figure S12. Secondary structures of the transfer RNAs in the mitogenome of Peltonotellus sp. Dashes and dots indicate Watson–Crick [file peerj-09-12465-s001.zip › supplementary materials -figure and table/Figure S22.tif]

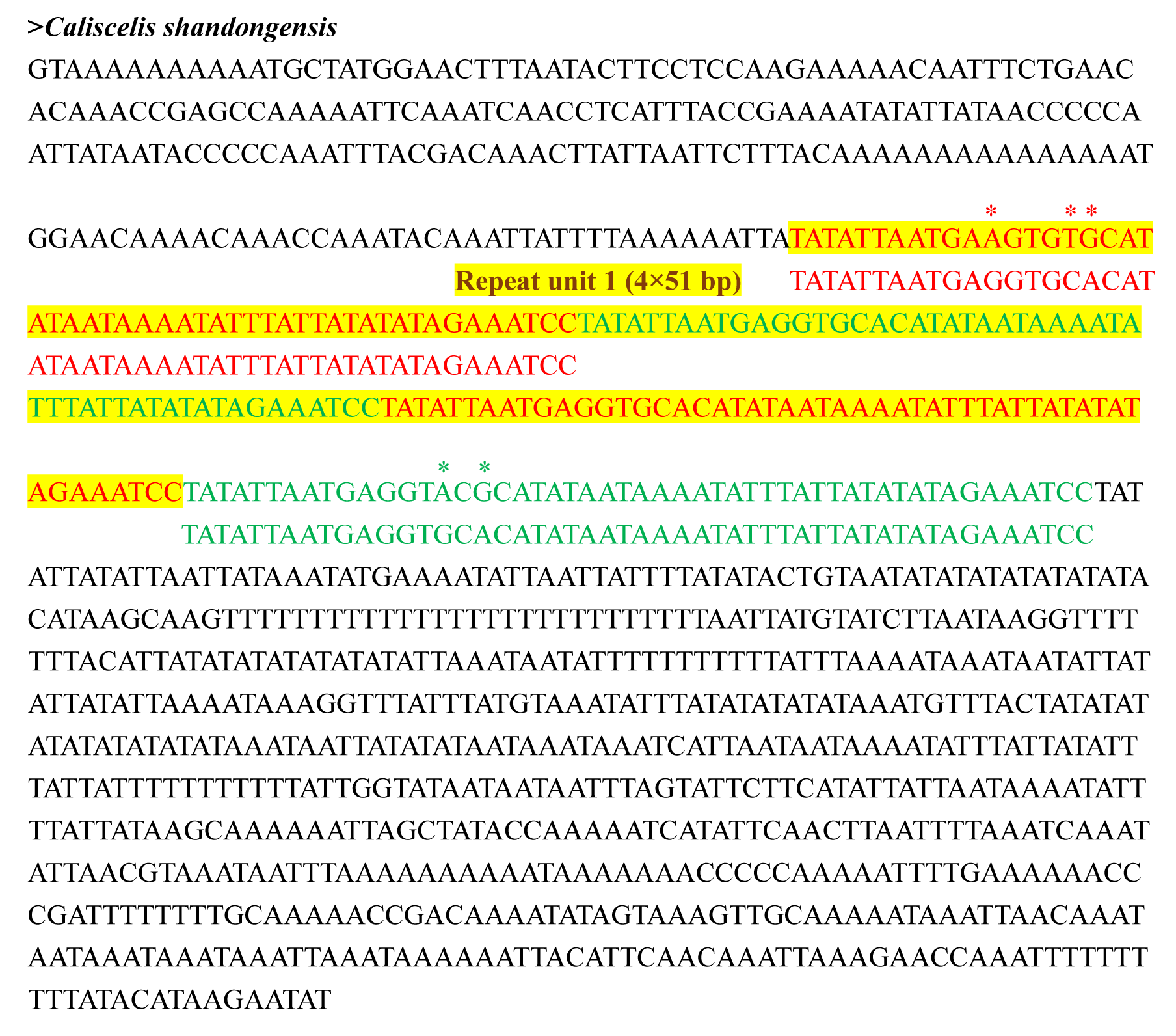

Supplement: Supplemental Information 1 — Figure S1. Secondary structures of the transfer RNAs in the mitogenome of Augilina tetraina. Dashes and dots indicate Watson–Crick and GU base pairing, respectively. Figure S2. Secondary structures of the transfer RNAs in the mitogenome of Augilina triaina. Dashes and dots indicate Watson–Crick and GU base pairing, respectively. Figure S3. Secondary structures of the transfer RNAs in the mitogenome of Symplana brevistrata. Dashes and dots indicate Watson–Crick and GU base pairing, respectively. Figure S4. Secondary structures of the transfer RNAs in the mitogenome of Symplana lii. Dashes and dots indicate Watson–Crick and GU base pairing, respectively. Figure S5. Secondary structures of the transfer RNAs in the mitogenome of Neosymplana vittatum. Dashes and dots indicate Watson–Crick and GU base pairing, respectively. Figure S6. Secondary structures of the transfer RNAs in the mitogenome of Pseudosymplanella nigrifasciata. Dashes and dots indicate Watson–Crick and GU base pairing, respectively. Figure S7. Secondary structures of the transfer RNAs in the mitogenome of Symplanella brevicephala. Dashes and dots indicate Watson–Crick and GU base pairing, respectively. Figure S8. Secondary structures of the transfer RNAs in the mitogenome of Symplanella unipuncta. Dashes and dots indicate Watson–Crick and GU base pairing, respectively. Figure S9. Secondary structures of the transfer RNAs in the mitogenome of Augilodes binghami. Dashes and dots indicate Watson–Crick and GU base pairing, respectively. Figure S10. Secondary structures of the transfer RNAs in the mitogenome of Cylindratus longicephalus. Dashes and dots indicate Watson–Crick and GU base pairing, respectively. Figure S11. Secondary structures of the transfer RNAs in the mitogenome of Caliscelis shandongensis. Dashes and dots indicate Watson–Crick and GU base pairing, respectively. Figure S12. Secondary structures of the transfer RNAs in the mitogenome of Peltonotellus sp. Dashes and dots indicate Watson–Crick [file peerj-09-12465-s001.zip › supplementary materials -figure and table/Figure S23.tif]

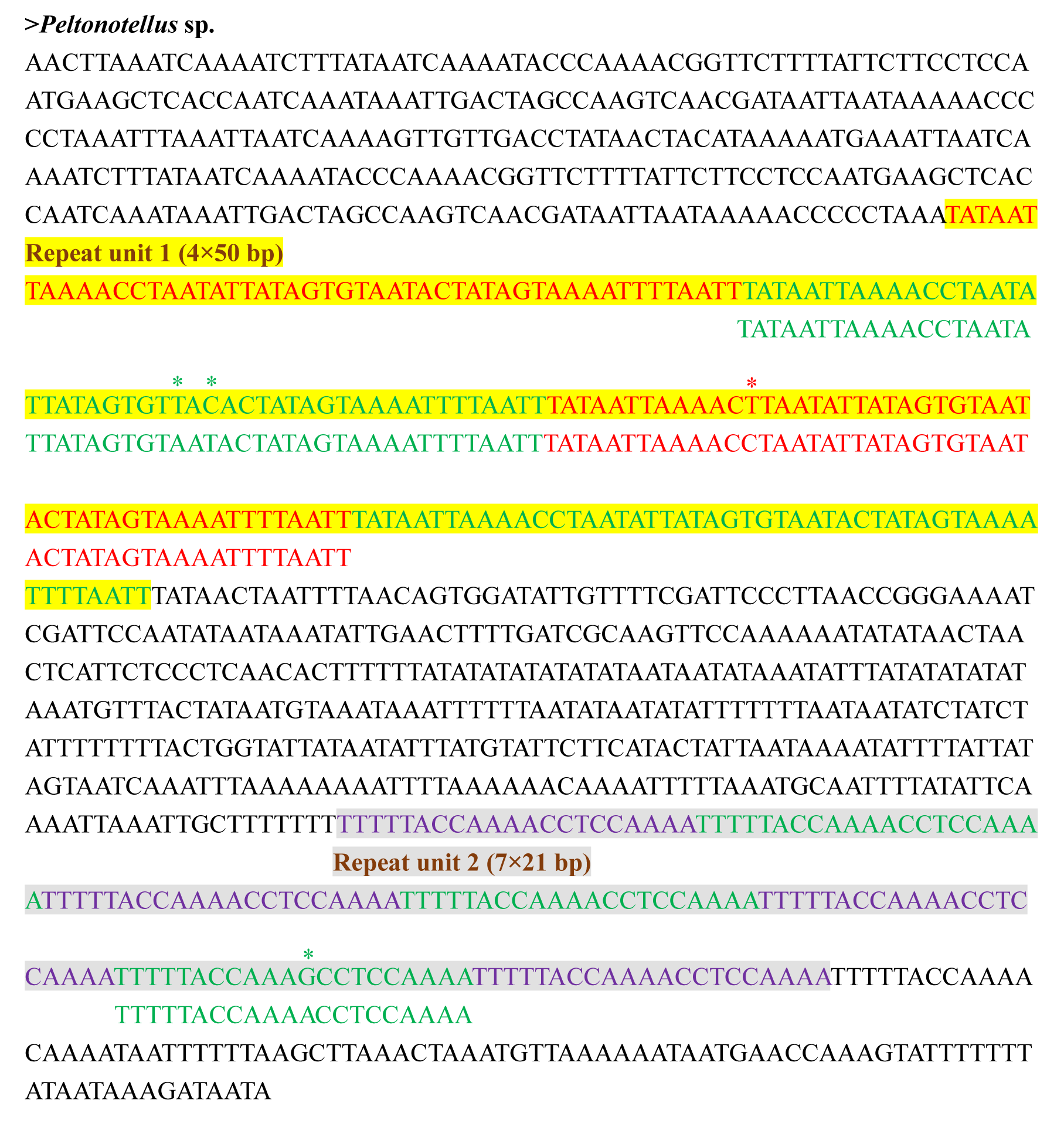

Supplement: Supplemental Information 1 — Figure S1. Secondary structures of the transfer RNAs in the mitogenome of Augilina tetraina. Dashes and dots indicate Watson–Crick and GU base pairing, respectively. Figure S2. Secondary structures of the transfer RNAs in the mitogenome of Augilina triaina. Dashes and dots indicate Watson–Crick and GU base pairing, respectively. Figure S3. Secondary structures of the transfer RNAs in the mitogenome of Symplana brevistrata. Dashes and dots indicate Watson–Crick and GU base pairing, respectively. Figure S4. Secondary structures of the transfer RNAs in the mitogenome of Symplana lii. Dashes and dots indicate Watson–Crick and GU base pairing, respectively. Figure S5. Secondary structures of the transfer RNAs in the mitogenome of Neosymplana vittatum. Dashes and dots indicate Watson–Crick and GU base pairing, respectively. Figure S6. Secondary structures of the transfer RNAs in the mitogenome of Pseudosymplanella nigrifasciata. Dashes and dots indicate Watson–Crick and GU base pairing, respectively. Figure S7. Secondary structures of the transfer RNAs in the mitogenome of Symplanella brevicephala. Dashes and dots indicate Watson–Crick and GU base pairing, respectively. Figure S8. Secondary structures of the transfer RNAs in the mitogenome of Symplanella unipuncta. Dashes and dots indicate Watson–Crick and GU base pairing, respectively. Figure S9. Secondary structures of the transfer RNAs in the mitogenome of Augilodes binghami. Dashes and dots indicate Watson–Crick and GU base pairing, respectively. Figure S10. Secondary structures of the transfer RNAs in the mitogenome of Cylindratus longicephalus. Dashes and dots indicate Watson–Crick and GU base pairing, respectively. Figure S11. Secondary structures of the transfer RNAs in the mitogenome of Caliscelis shandongensis. Dashes and dots indicate Watson–Crick and GU base pairing, respectively. Figure S12. Secondary structures of the transfer RNAs in the mitogenome of Peltonotellus sp. Dashes and dots indicate Watson–Crick [file peerj-09-12465-s001.zip › supplementary materials -figure and table/Figure S24.tif]

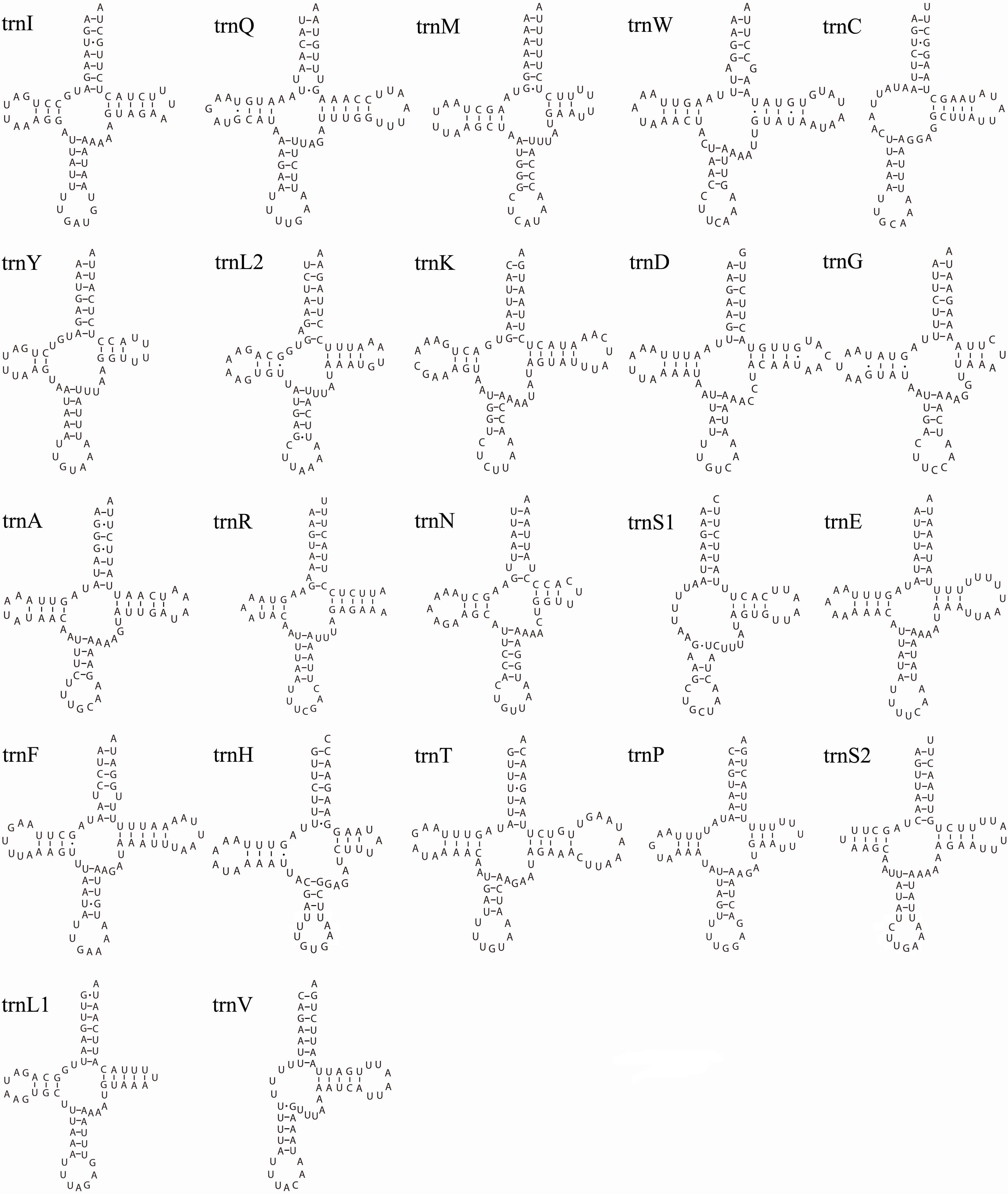

Supplement: Supplemental Information 1 — Figure S1. Secondary structures of the transfer RNAs in the mitogenome of Augilina tetraina. Dashes and dots indicate Watson–Crick and GU base pairing, respectively. Figure S2. Secondary structures of the transfer RNAs in the mitogenome of Augilina triaina. Dashes and dots indicate Watson–Crick and GU base pairing, respectively. Figure S3. Secondary structures of the transfer RNAs in the mitogenome of Symplana brevistrata. Dashes and dots indicate Watson–Crick and GU base pairing, respectively. Figure S4. Secondary structures of the transfer RNAs in the mitogenome of Symplana lii. Dashes and dots indicate Watson–Crick and GU base pairing, respectively. Figure S5. Secondary structures of the transfer RNAs in the mitogenome of Neosymplana vittatum. Dashes and dots indicate Watson–Crick and GU base pairing, respectively. Figure S6. Secondary structures of the transfer RNAs in the mitogenome of Pseudosymplanella nigrifasciata. Dashes and dots indicate Watson–Crick and GU base pairing, respectively. Figure S7. Secondary structures of the transfer RNAs in the mitogenome of Symplanella brevicephala. Dashes and dots indicate Watson–Crick and GU base pairing, respectively. Figure S8. Secondary structures of the transfer RNAs in the mitogenome of Symplanella unipuncta. Dashes and dots indicate Watson–Crick and GU base pairing, respectively. Figure S9. Secondary structures of the transfer RNAs in the mitogenome of Augilodes binghami. Dashes and dots indicate Watson–Crick and GU base pairing, respectively. Figure S10. Secondary structures of the transfer RNAs in the mitogenome of Cylindratus longicephalus. Dashes and dots indicate Watson–Crick and GU base pairing, respectively. Figure S11. Secondary structures of the transfer RNAs in the mitogenome of Caliscelis shandongensis. Dashes and dots indicate Watson–Crick and GU base pairing, respectively. Figure S12. Secondary structures of the transfer RNAs in the mitogenome of Peltonotellus sp. Dashes and dots indicate Watson–Crick [file peerj-09-12465-s001.zip › supplementary materials -figure and table/Figure S3.jpg]

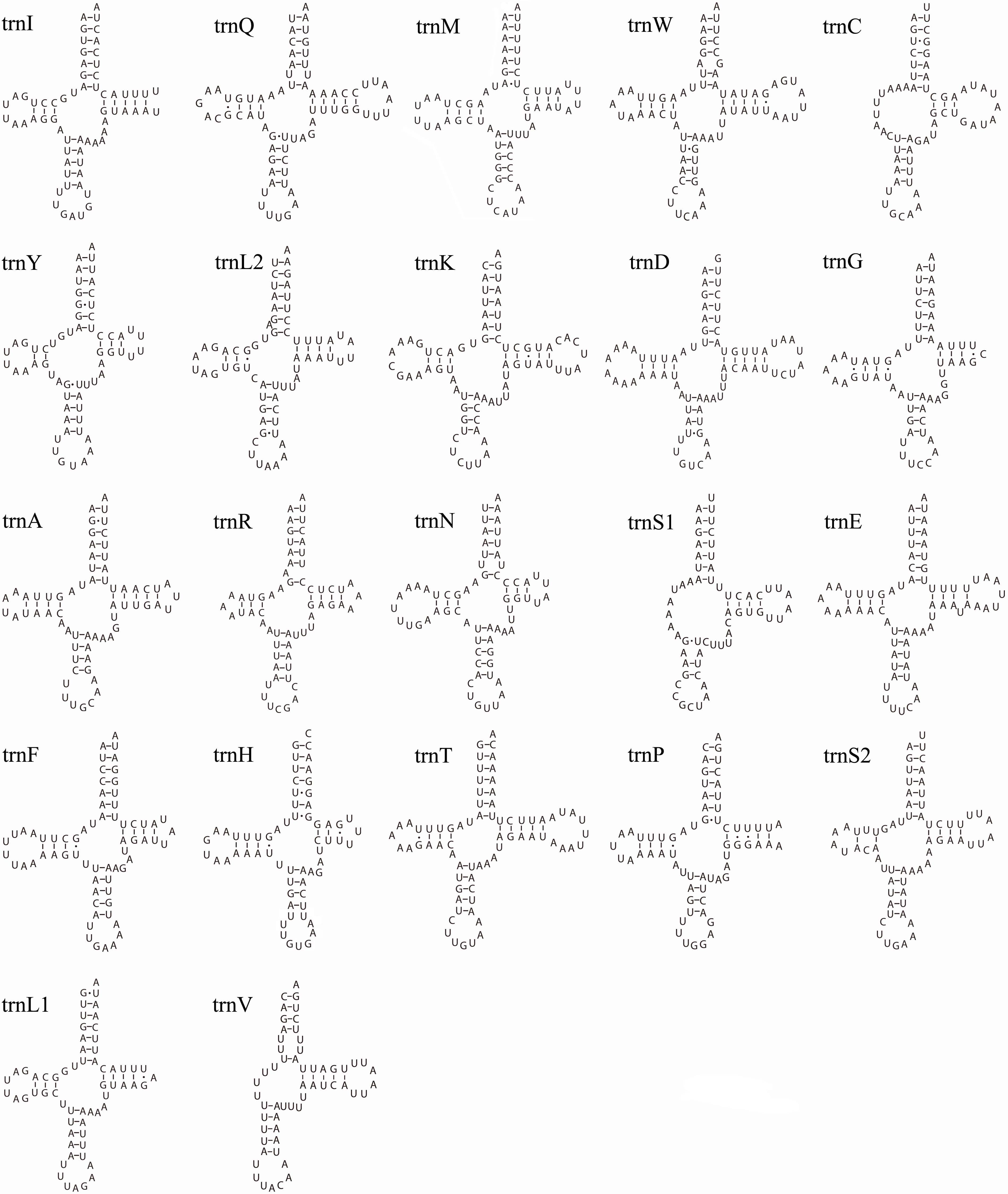

Supplement: Supplemental Information 1 — Figure S1. Secondary structures of the transfer RNAs in the mitogenome of Augilina tetraina. Dashes and dots indicate Watson–Crick and GU base pairing, respectively. Figure S2. Secondary structures of the transfer RNAs in the mitogenome of Augilina triaina. Dashes and dots indicate Watson–Crick and GU base pairing, respectively. Figure S3. Secondary structures of the transfer RNAs in the mitogenome of Symplana brevistrata. Dashes and dots indicate Watson–Crick and GU base pairing, respectively. Figure S4. Secondary structures of the transfer RNAs in the mitogenome of Symplana lii. Dashes and dots indicate Watson–Crick and GU base pairing, respectively. Figure S5. Secondary structures of the transfer RNAs in the mitogenome of Neosymplana vittatum. Dashes and dots indicate Watson–Crick and GU base pairing, respectively. Figure S6. Secondary structures of the transfer RNAs in the mitogenome of Pseudosymplanella nigrifasciata. Dashes and dots indicate Watson–Crick and GU base pairing, respectively. Figure S7. Secondary structures of the transfer RNAs in the mitogenome of Symplanella brevicephala. Dashes and dots indicate Watson–Crick and GU base pairing, respectively. Figure S8. Secondary structures of the transfer RNAs in the mitogenome of Symplanella unipuncta. Dashes and dots indicate Watson–Crick and GU base pairing, respectively. Figure S9. Secondary structures of the transfer RNAs in the mitogenome of Augilodes binghami. Dashes and dots indicate Watson–Crick and GU base pairing, respectively. Figure S10. Secondary structures of the transfer RNAs in the mitogenome of Cylindratus longicephalus. Dashes and dots indicate Watson–Crick and GU base pairing, respectively. Figure S11. Secondary structures of the transfer RNAs in the mitogenome of Caliscelis shandongensis. Dashes and dots indicate Watson–Crick and GU base pairing, respectively. Figure S12. Secondary structures of the transfer RNAs in the mitogenome of Peltonotellus sp. Dashes and dots indicate Watson–Crick [file peerj-09-12465-s001.zip › supplementary materials -figure and table/Figure S4.jpg]

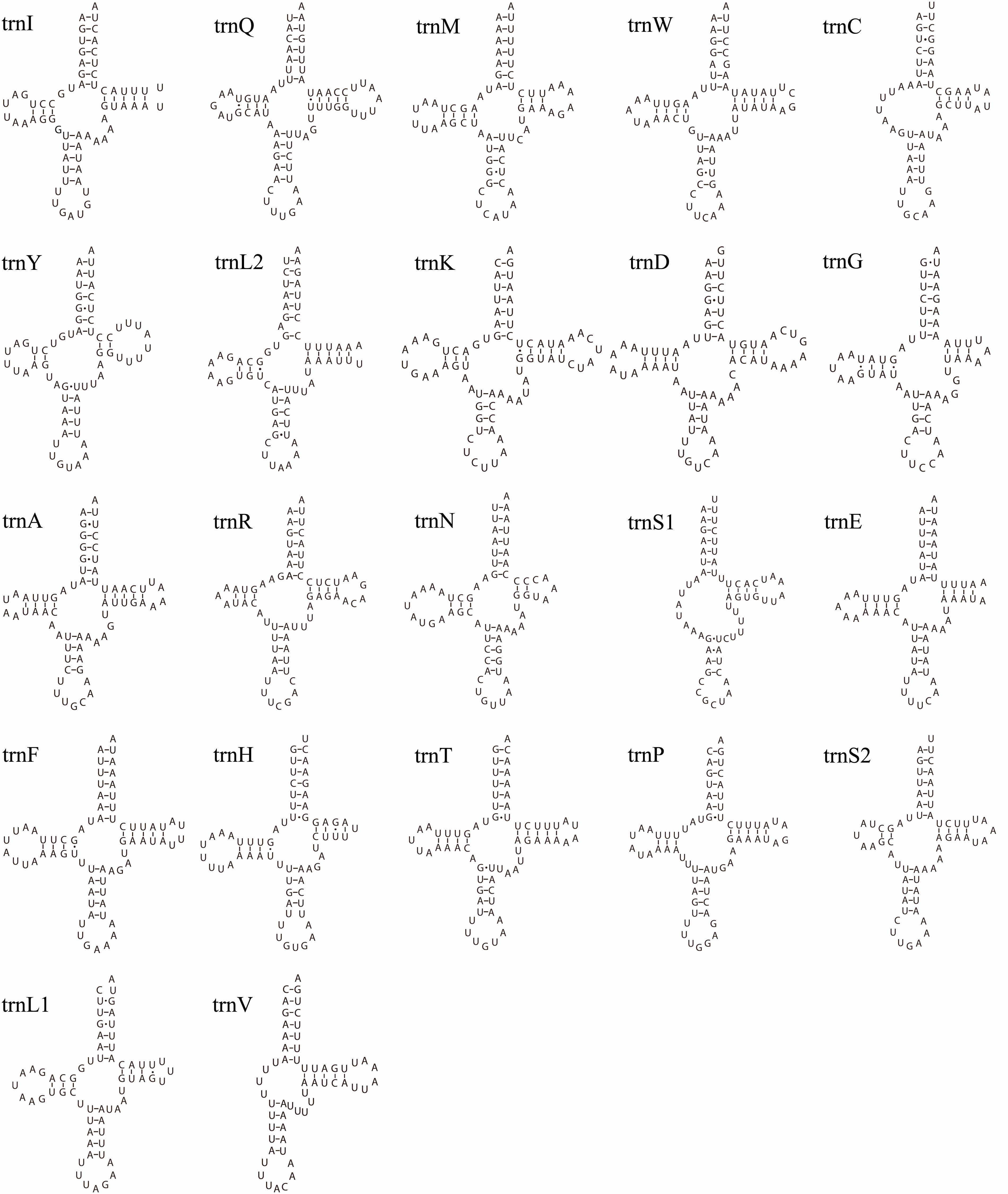

Supplement: Supplemental Information 1 — Figure S1. Secondary structures of the transfer RNAs in the mitogenome of Augilina tetraina. Dashes and dots indicate Watson–Crick and GU base pairing, respectively. Figure S2. Secondary structures of the transfer RNAs in the mitogenome of Augilina triaina. Dashes and dots indicate Watson–Crick and GU base pairing, respectively. Figure S3. Secondary structures of the transfer RNAs in the mitogenome of Symplana brevistrata. Dashes and dots indicate Watson–Crick and GU base pairing, respectively. Figure S4. Secondary structures of the transfer RNAs in the mitogenome of Symplana lii. Dashes and dots indicate Watson–Crick and GU base pairing, respectively. Figure S5. Secondary structures of the transfer RNAs in the mitogenome of Neosymplana vittatum. Dashes and dots indicate Watson–Crick and GU base pairing, respectively. Figure S6. Secondary structures of the transfer RNAs in the mitogenome of Pseudosymplanella nigrifasciata. Dashes and dots indicate Watson–Crick and GU base pairing, respectively. Figure S7. Secondary structures of the transfer RNAs in the mitogenome of Symplanella brevicephala. Dashes and dots indicate Watson–Crick and GU base pairing, respectively. Figure S8. Secondary structures of the transfer RNAs in the mitogenome of Symplanella unipuncta. Dashes and dots indicate Watson–Crick and GU base pairing, respectively. Figure S9. Secondary structures of the transfer RNAs in the mitogenome of Augilodes binghami. Dashes and dots indicate Watson–Crick and GU base pairing, respectively. Figure S10. Secondary structures of the transfer RNAs in the mitogenome of Cylindratus longicephalus. Dashes and dots indicate Watson–Crick and GU base pairing, respectively. Figure S11. Secondary structures of the transfer RNAs in the mitogenome of Caliscelis shandongensis. Dashes and dots indicate Watson–Crick and GU base pairing, respectively. Figure S12. Secondary structures of the transfer RNAs in the mitogenome of Peltonotellus sp. Dashes and dots indicate Watson–Crick [file peerj-09-12465-s001.zip › supplementary materials -figure and table/Figure S5.jpg]

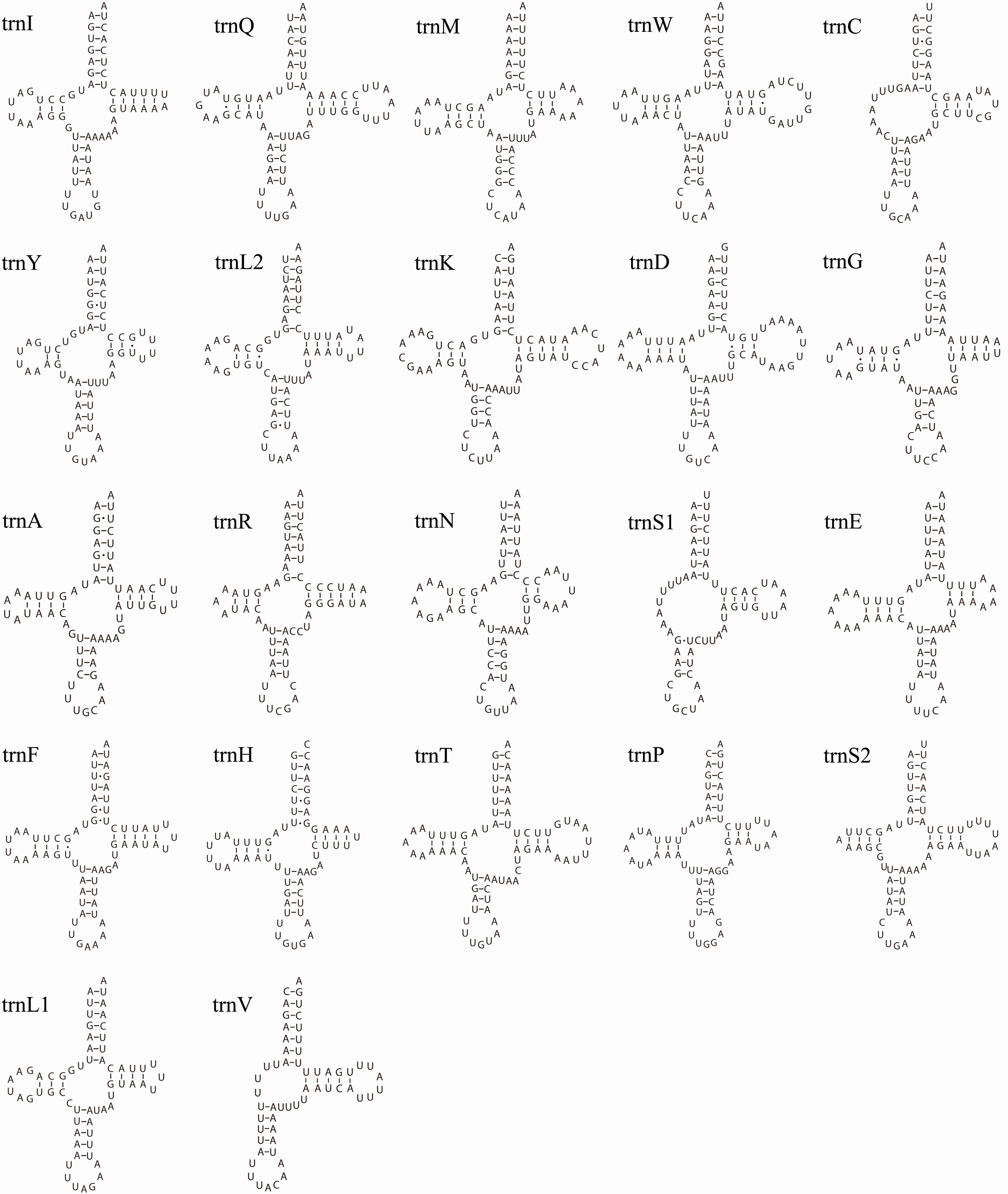

Supplement: Supplemental Information 1 — Figure S1. Secondary structures of the transfer RNAs in the mitogenome of Augilina tetraina. Dashes and dots indicate Watson–Crick and GU base pairing, respectively. Figure S2. Secondary structures of the transfer RNAs in the mitogenome of Augilina triaina. Dashes and dots indicate Watson–Crick and GU base pairing, respectively. Figure S3. Secondary structures of the transfer RNAs in the mitogenome of Symplana brevistrata. Dashes and dots indicate Watson–Crick and GU base pairing, respectively. Figure S4. Secondary structures of the transfer RNAs in the mitogenome of Symplana lii. Dashes and dots indicate Watson–Crick and GU base pairing, respectively. Figure S5. Secondary structures of the transfer RNAs in the mitogenome of Neosymplana vittatum. Dashes and dots indicate Watson–Crick and GU base pairing, respectively. Figure S6. Secondary structures of the transfer RNAs in the mitogenome of Pseudosymplanella nigrifasciata. Dashes and dots indicate Watson–Crick and GU base pairing, respectively. Figure S7. Secondary structures of the transfer RNAs in the mitogenome of Symplanella brevicephala. Dashes and dots indicate Watson–Crick and GU base pairing, respectively. Figure S8. Secondary structures of the transfer RNAs in the mitogenome of Symplanella unipuncta. Dashes and dots indicate Watson–Crick and GU base pairing, respectively. Figure S9. Secondary structures of the transfer RNAs in the mitogenome of Augilodes binghami. Dashes and dots indicate Watson–Crick and GU base pairing, respectively. Figure S10. Secondary structures of the transfer RNAs in the mitogenome of Cylindratus longicephalus. Dashes and dots indicate Watson–Crick and GU base pairing, respectively. Figure S11. Secondary structures of the transfer RNAs in the mitogenome of Caliscelis shandongensis. Dashes and dots indicate Watson–Crick and GU base pairing, respectively. Figure S12. Secondary structures of the transfer RNAs in the mitogenome of Peltonotellus sp. Dashes and dots indicate Watson–Crick [file peerj-09-12465-s001.zip › supplementary materials -figure and table/Figure S6.jpg]

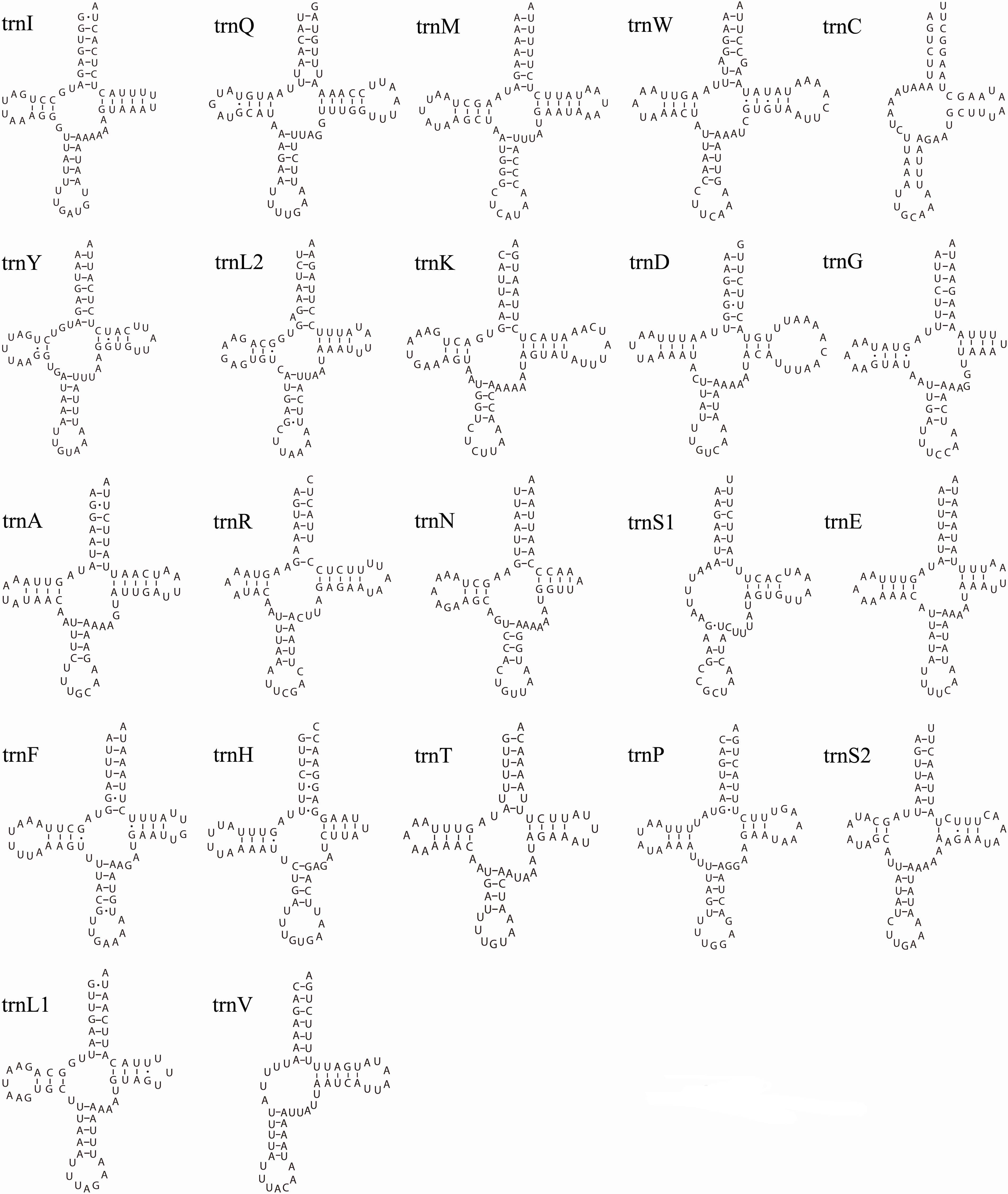

Supplement: Supplemental Information 1 — Figure S1. Secondary structures of the transfer RNAs in the mitogenome of Augilina tetraina. Dashes and dots indicate Watson–Crick and GU base pairing, respectively. Figure S2. Secondary structures of the transfer RNAs in the mitogenome of Augilina triaina. Dashes and dots indicate Watson–Crick and GU base pairing, respectively. Figure S3. Secondary structures of the transfer RNAs in the mitogenome of Symplana brevistrata. Dashes and dots indicate Watson–Crick and GU base pairing, respectively. Figure S4. Secondary structures of the transfer RNAs in the mitogenome of Symplana lii. Dashes and dots indicate Watson–Crick and GU base pairing, respectively. Figure S5. Secondary structures of the transfer RNAs in the mitogenome of Neosymplana vittatum. Dashes and dots indicate Watson–Crick and GU base pairing, respectively. Figure S6. Secondary structures of the transfer RNAs in the mitogenome of Pseudosymplanella nigrifasciata. Dashes and dots indicate Watson–Crick and GU base pairing, respectively. Figure S7. Secondary structures of the transfer RNAs in the mitogenome of Symplanella brevicephala. Dashes and dots indicate Watson–Crick and GU base pairing, respectively. Figure S8. Secondary structures of the transfer RNAs in the mitogenome of Symplanella unipuncta. Dashes and dots indicate Watson–Crick and GU base pairing, respectively. Figure S9. Secondary structures of the transfer RNAs in the mitogenome of Augilodes binghami. Dashes and dots indicate Watson–Crick and GU base pairing, respectively. Figure S10. Secondary structures of the transfer RNAs in the mitogenome of Cylindratus longicephalus. Dashes and dots indicate Watson–Crick and GU base pairing, respectively. Figure S11. Secondary structures of the transfer RNAs in the mitogenome of Caliscelis shandongensis. Dashes and dots indicate Watson–Crick and GU base pairing, respectively. Figure S12. Secondary structures of the transfer RNAs in the mitogenome of Peltonotellus sp. Dashes and dots indicate Watson–Crick [file peerj-09-12465-s001.zip › supplementary materials -figure and table/Figure S7.jpg]

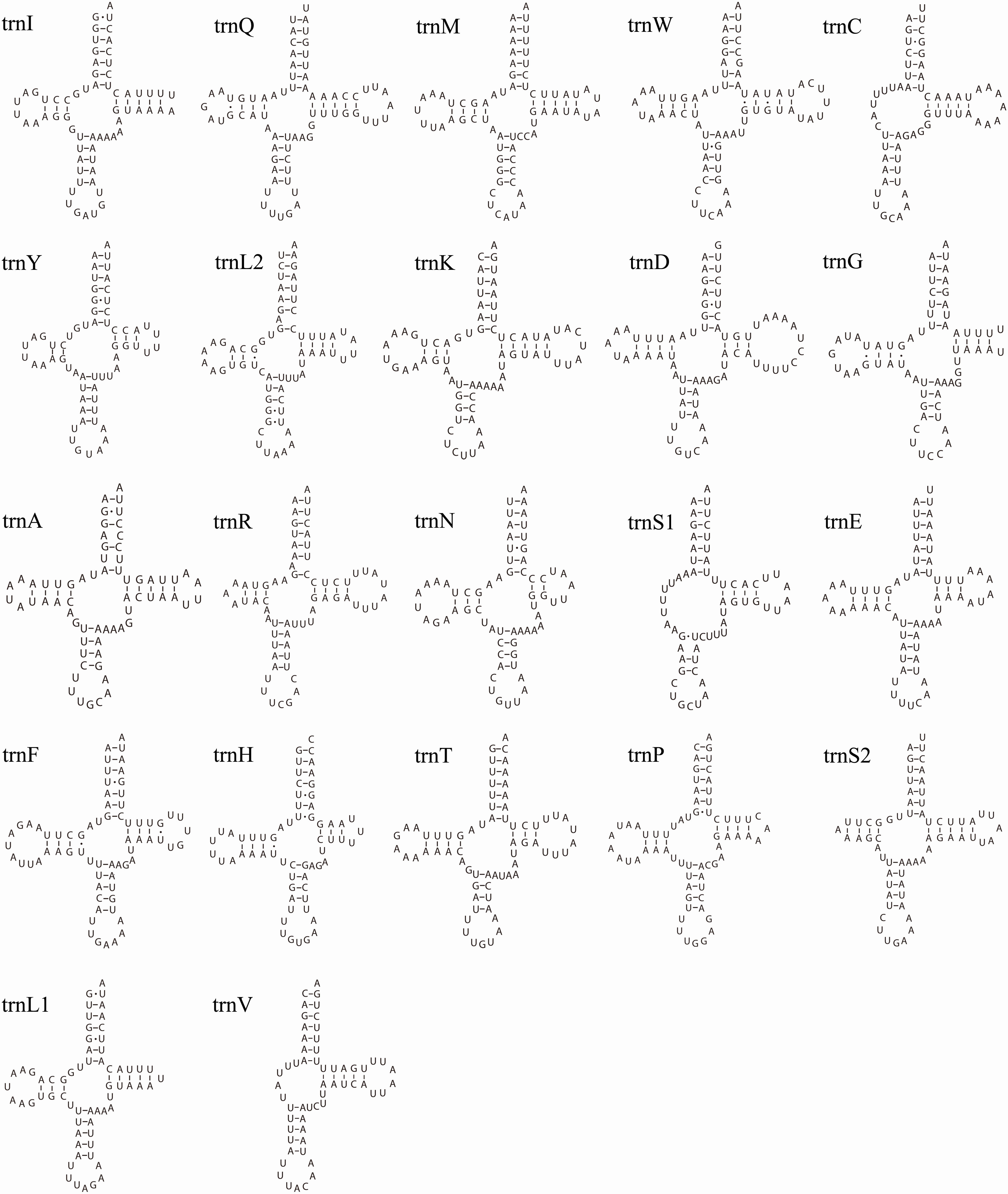

Supplement: Supplemental Information 1 — Figure S1. Secondary structures of the transfer RNAs in the mitogenome of Augilina tetraina. Dashes and dots indicate Watson–Crick and GU base pairing, respectively. Figure S2. Secondary structures of the transfer RNAs in the mitogenome of Augilina triaina. Dashes and dots indicate Watson–Crick and GU base pairing, respectively. Figure S3. Secondary structures of the transfer RNAs in the mitogenome of Symplana brevistrata. Dashes and dots indicate Watson–Crick and GU base pairing, respectively. Figure S4. Secondary structures of the transfer RNAs in the mitogenome of Symplana lii. Dashes and dots indicate Watson–Crick and GU base pairing, respectively. Figure S5. Secondary structures of the transfer RNAs in the mitogenome of Neosymplana vittatum. Dashes and dots indicate Watson–Crick and GU base pairing, respectively. Figure S6. Secondary structures of the transfer RNAs in the mitogenome of Pseudosymplanella nigrifasciata. Dashes and dots indicate Watson–Crick and GU base pairing, respectively. Figure S7. Secondary structures of the transfer RNAs in the mitogenome of Symplanella brevicephala. Dashes and dots indicate Watson–Crick and GU base pairing, respectively. Figure S8. Secondary structures of the transfer RNAs in the mitogenome of Symplanella unipuncta. Dashes and dots indicate Watson–Crick and GU base pairing, respectively. Figure S9. Secondary structures of the transfer RNAs in the mitogenome of Augilodes binghami. Dashes and dots indicate Watson–Crick and GU base pairing, respectively. Figure S10. Secondary structures of the transfer RNAs in the mitogenome of Cylindratus longicephalus. Dashes and dots indicate Watson–Crick and GU base pairing, respectively. Figure S11. Secondary structures of the transfer RNAs in the mitogenome of Caliscelis shandongensis. Dashes and dots indicate Watson–Crick and GU base pairing, respectively. Figure S12. Secondary structures of the transfer RNAs in the mitogenome of Peltonotellus sp. Dashes and dots indicate Watson–Crick [file peerj-09-12465-s001.zip › supplementary materials -figure and table/Figure S8.jpg]

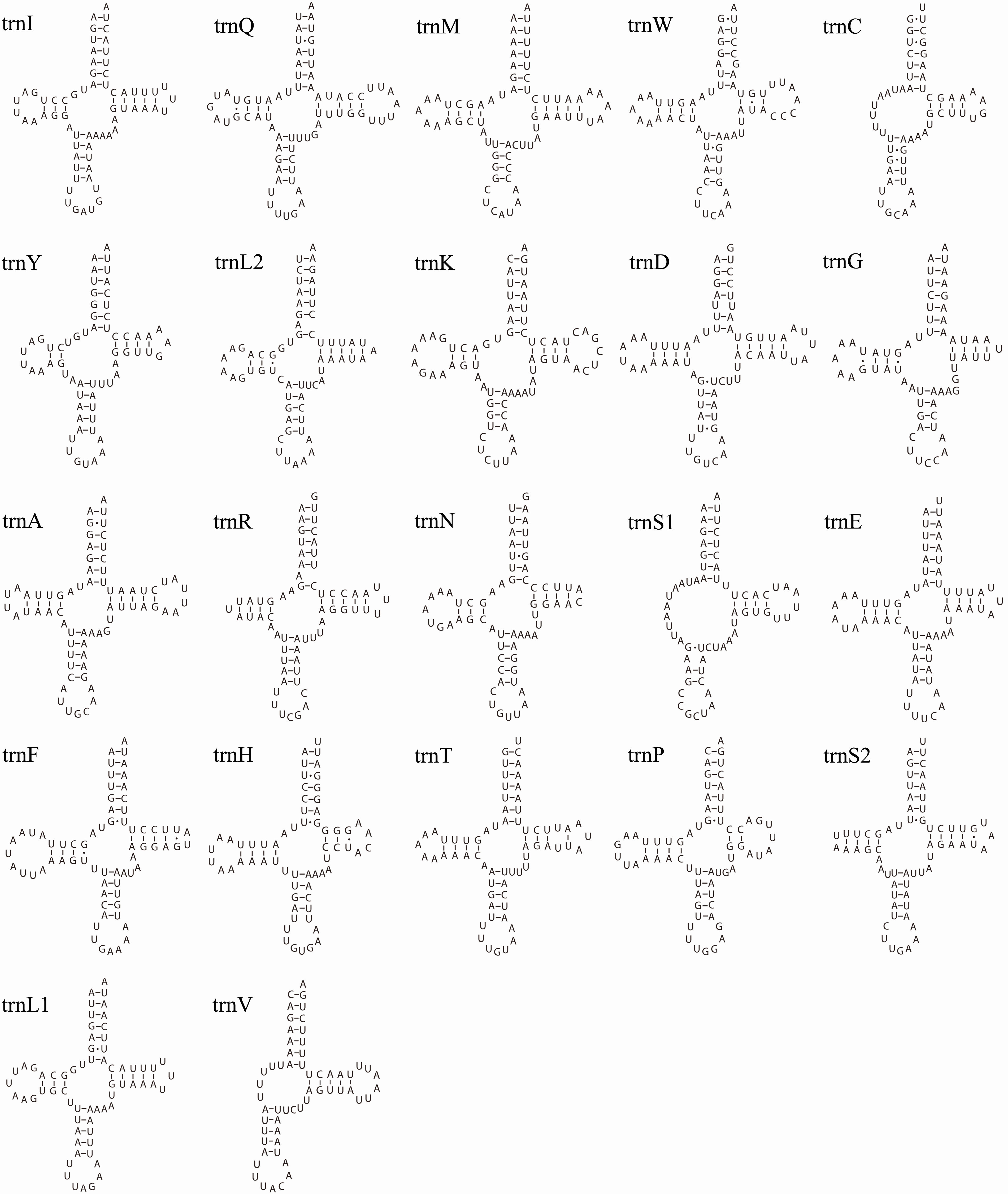

Supplement: Supplemental Information 1 — Figure S1. Secondary structures of the transfer RNAs in the mitogenome of Augilina tetraina. Dashes and dots indicate Watson–Crick and GU base pairing, respectively. Figure S2. Secondary structures of the transfer RNAs in the mitogenome of Augilina triaina. Dashes and dots indicate Watson–Crick and GU base pairing, respectively. Figure S3. Secondary structures of the transfer RNAs in the mitogenome of Symplana brevistrata. Dashes and dots indicate Watson–Crick and GU base pairing, respectively. Figure S4. Secondary structures of the transfer RNAs in the mitogenome of Symplana lii. Dashes and dots indicate Watson–Crick and GU base pairing, respectively. Figure S5. Secondary structures of the transfer RNAs in the mitogenome of Neosymplana vittatum. Dashes and dots indicate Watson–Crick and GU base pairing, respectively. Figure S6. Secondary structures of the transfer RNAs in the mitogenome of Pseudosymplanella nigrifasciata. Dashes and dots indicate Watson–Crick and GU base pairing, respectively. Figure S7. Secondary structures of the transfer RNAs in the mitogenome of Symplanella brevicephala. Dashes and dots indicate Watson–Crick and GU base pairing, respectively. Figure S8. Secondary structures of the transfer RNAs in the mitogenome of Symplanella unipuncta. Dashes and dots indicate Watson–Crick and GU base pairing, respectively. Figure S9. Secondary structures of the transfer RNAs in the mitogenome of Augilodes binghami. Dashes and dots indicate Watson–Crick and GU base pairing, respectively. Figure S10. Secondary structures of the transfer RNAs in the mitogenome of Cylindratus longicephalus. Dashes and dots indicate Watson–Crick and GU base pairing, respectively. Figure S11. Secondary structures of the transfer RNAs in the mitogenome of Caliscelis shandongensis. Dashes and dots indicate Watson–Crick and GU base pairing, respectively. Figure S12. Secondary structures of the transfer RNAs in the mitogenome of Peltonotellus sp. Dashes and dots indicate Watson–Crick [file peerj-09-12465-s001.zip › supplementary materials -figure and table/Figure S9.jpg]
